# PARP1-BCAT2 axis upregulates ABCG1 via histone lactylation to drive acquired PARP inhibitor resistance in prostate cancer

**DOI:** 10.1186/s13046-026-03719-1

**Published:** 2026-05-11

**Authors:** Wangli Mei, Hang Zhou, Weiyi Li, Yongqiang Liu, Chaozhi Tang, Mengyu Wei, Zhen Zhou, Bowen Ye, Hanwen Niu, Weitian Wang, Kaiqi Yang, Yue Zhang, Liqun Huang, Yang Yu, Xiaofei Wen, Lin Ye

**Affiliations:** 1 Department of Urology, Shanghai East Hospital, School of Medicine, Tongji University, Shanghai, 200120 China; 2 Department of Urology, Shanghai Geriatrics Medical Center, Zhongshan Hospital Fudan University Minhang Campus, Shanghai, 201104 China; 3 Department of Urology, Shanghai Tenth People’s Hospital, School of Medicine, Tongji University, Shanghai, 200072 China; 4 Department of Laboratory Medicine, Shanghai Changzheng Hospital, Naval Medical University, Shanghai, 200003 China

**Keywords:** BCAT2, PARP1, ABCG1, Lactylation, PARPi resistance, Prostate cancer

## Abstract

**Background:**

Poly (ADP-ribose) polymerase inhibitor (PARPi) resistance poses a significant challenge in prostate cancer (PCa). Although branched-chain amino acid (BCAA) metabolism is implicated in cancer biology, its specific role in PARPi resistance remains unclear. This study aims to investigate how BCAA metabolism contributes to PARPi resistance in PCa.

**Methods:**

We compared BCAA and Branched-Chain Amino Acid Aminotransferase 2 (BCAT2) levels between PARPi-resistant and PARPi-sensitive cell lines and assessed their clinical relevance. Functional studies were conducted in vitro and in vivo using cell and mouse models. Mechanistic assays, including RNA sequencing, metabolomics, RNA-binding protein immunoprecipitation (RIP), chromatin immunoprecipitation (ChIP), and Cleavage Under Targets and Tagmentation (CUT&Tag), were used to delineate BCAT2-mediated PARPi resistance.

**Results:**

BCAT2 expression correlated with PARPi resistance in PCa, and increased BCAA/BCAT2 levels in PARPi-resistant tissues were associated with reduced patient survival. Mechanistically, the DNA-binding domain (DBD) of PARP1 directly bound BCAT2 mRNA and regulated its stability; PARPi-induced PARP1 trapping weakened this interaction, increased BCAT2 expression, and promoted resistance. Transcriptomic and energy-metabolism analyses indicated that BCAT2 enhanced ABCG1 transcription by augmenting glycolysis and lactate secretion, thereby increasing histone H3K18la lactylation. These findings support a PARP1–BCAT2–ABCG1 axis in PARPi resistance. Combining a BCAT2 inhibitor with PARPi produced synergistic effects in cell line–derived xenografts (CDXs) and patient-derived organoids (PDOs).

**Conclusion:**

The PARP1–BCAT2/H3K18la–ABCG1 axis drives PARPi resistance in PCa. Targeted BCAT2 inhibition may enhance the therapeutic efficacy of PARPi.

**Supplementary Information:**

The online version contains supplementary material available at 10.1186/s13046-026-03719-1.

## Background

Prostate cancer (PCa) is the most frequently diagnosed malignancy and the second leading cause of cancer-related death in men worldwide [1]. Although the primary tumor can often be managed with surgery or radiotherapy, advanced PCa remains a therapeutic challenge owing to marked heterogeneity and evolutionary capacity [2, 3]. Although current treatments may extend survival, they often cause substantial toxicities, reduce the quality of life, and rarely achieve a cure [4]; treatment options also become limited in later disease stages [5].

Recently approved PARP inhibitors (PARPi), including olaparib, talazoparib, rucaparib, and niraparib, offer a targeted strategy for metastatic castration-resistant PCa, particularly in tumors with homologous recombination repair gene alterations [6–8]. Despite improvements with PARPi maintenance therapy, acquired resistance commonly emerges after the initial response, limiting long-term benefit [9, 10]. Established mechanisms include restoration of homologous recombination via BRCA reversion mutations, increased drug efflux, and adaptations in DNA damage–response (DDR) pathways [11–13]. Defining additional resistance mechanisms and developing rational combinations are therefore urgent priorities.

Cancer cells reprogram metabolism to sustain proliferation, evade cell death, and adapt to hostile microenvironments by supplying bioenergy and biosynthetic precursors and reducing equivalents [14–18]. This plasticity fuels growth and promotes therapy resistance [19, 20]. Among metabolic pathways, branched-chain amino acid (BCAA) metabolism has gained attention for its roles in tumor progression and treatment resistance [21, 22]. Through catabolism, BCAAs replenish tricarboxylic acid (TCA) cycle intermediates and contribute to lactate production [22]. Lactate, in turn, serves as a direct substrate for histone lactylation, an epigenetic modification that links glycolytic flux to chromatin state and transcriptional reprogramming [23–25].

BCAT2, a key enzyme catalyzing the first step of BCAA transamination, has been implicated in several malignancies, including PCa and pancreatic ductal adenocarcinoma [26]. Notably, BCAT2 knockdown or a low-BCAA diet can suppress tumor progression [27, 28]. By driving BCAA catabolism, BCAT2 may increase lactate availability for histone lactylation, potentially linking metabolism to epigenetic regulation and drug resistance. In therapy resistance, ATP-binding cassette transporters, including ABCG family members, are critically involved in efflux processes [29, 30].

In this study, we used PARPi-resistant cell lines, clinical specimen analyses, and transcriptomic screening to identify the BCAT2/BCAA pathway as a mediator of resistance. We further identified and preliminarily validated a non-canonical function of PARP1 as an RNA-binding protein (RBP) that regulates BCAT2 mRNA stability [31]. Mechanistically, we delineate a signaling axis in which BCAT2/BCAA metabolism promotes lactate production, facilitates histone H3K18 lactylation, recruits the transcription factor KLF3, and activates transcription of ABCG1, thereby driving PARPi resistance. Using preclinical models, we show that targeted BCAT2 inhibition restores PARPi sensitivity, supporting its potential as a combination therapy target.

## Methods

### Clinical sample collection

A total of 6 paired tissue samples from PARPi-sensitive and PARPi-resistant PCa cases were obtained for RNA and protein extraction from Shanghai East Hospital, Tongji University (Shanghai, China), with all specimens confirmed by an experienced pathologist. Additionally, 516 patients with PCa who underwent radical prostatectomy were enrolled for IHC staining or PDO culture in this study. The research was conducted in accordance with the Declaration of Helsinki and received ethical approval from the Ethical Committee of Shanghai East Hospital (Approval number: 2024YS-244). Written informed consent was obtained from all participating patients.

### Cell culture

PCa cell lines (DU145, PC3, LNCaP), and HEK293T cells were obtained from the Chinese Academy of Sciences Committee Typical Culture Collection Cell Bank (Shanghai, China). HEK293T cells were maintained in Dulbecco’s modified Eagle medium (DMEM; Gibco), and PCa cell lines in Roswell Park Memorial Institute 1640 medium (Gibco). Media for HEK293T and PCa cells contained 10% fetal bovine serum (FBS; Gibco) and 1% penicillin–streptomycin (Gibco). Especially, LNCaP cells were maintained in androgen-replete conditions using 10% FBS without charcoal-stripped serum (CSS) throughout the experiments, as previous studies indicate that HR-proficient LNCaP cells exhibit better responses to PARP inhibitors in the presence of physiological androgen levels [50]. All cells were maintained at 37 °C in a humidified incubator with 5% CO₂/95% air.

### Construction of olaparib-resistant cell lines

As DU145 is androgen receptor-negative, it represents the castration-resistant stage of prostate cancer, which is clinically relevant for studying PARP inhibitor resistance in advanced disease where AR-targeted therapies have failed. Therefore, DU145 was chosen to establish stable drug-resistant cells of olaparib. Cells were exposed to progressively increasing concentrations of olaparib (1–40 µM) to induce acquired resistance. After ~ 6 months of continuous culture in drug-containing medium, olaparib-resistant cells were established. Resistant cells were routinely maintained in medium supplemented with a low concentration of olaparib, which was withdrawn prior to experimental assays.

### Determination of 50% inhibitory concentration (IC_50_)

IC₅₀ values for olaparib (AZD2281), talazoparib (BMN-673), rucaparib (AG014699), and niraparib (MK-4827) (all from MedChemExpress, Monmouth Junction, NJ, USA) were determined using the Cell Counting Kit-8 (CCK-8; Epizyme, Shanghai, China). Cells (5 × 10³ per well) were seeded in 96-well plates, allowed to adhere overnight, and then treated with serial concentrations of each inhibitor or DMSO (negative control, final concentration ≤ 0.1%, MedChemExpress) for 5 days. Cell survival rates were normalized to DMSO vehicle control. Absorbance at 450 nm was measured on a microplate reader (SpectraMax iD5; Molecular Devices, San Jose, CA, USA). IC₅₀ values were calculated using GraphPad Prism (version 9.0).

### Drug synergy assay

The BCAT2 inhibitor BCAT-IN-2 and the PARP inhibitor olaparib (MedChemExpress) were used for combination testing. Cells were seeded in 96-well plates (5,000 cells/well), allowed to adhere overnight, and treated with dose matrices of the two agents for 5 days. Cell viability was assessed using CCK-8 (absorbance at 450 nm). All treatments were performed in triplicate. Drug interaction was quantified using CompuSyn or SynergyFinder software; a CI value < 1 indicates synergy, CI = 1 additivity, and CI > 1 antagonism.

### Colony-formation assay

PC3 and DU145 cells were seeded at a density of 1 × 10³ cells per well in 6-well plates and allowed to adhere for 24 h prior to drug treatment. The cells were then cultured for 10–14 days until colonies formed. Due to the poor adhesion and slower growth rate of LNCaP cells, they were seeded at a higher density of 1.5 × 10³ cells per well and allowed to adhere before drug treatment. These cells were cultured for an extended period of 14–21 days until colonies formed. LNCaP cells were cultured in standard 2D monolayer conditions, not 3D Matrigel or soft agar, to maintain consistency with DU145 and PC3 comparisons. Colonies were fixed with 4% paraformaldehyde (Servicebio, Wuhan, China) for 30 min at room temperature and stained with 0.5% crystal violet (Beyotime, Shanghai, China) for 10 min. Colonies were imaged and counted using ImageJ.

### RNA sequencing (RNA-seq)

Total RNA was extracted using TRIzol reagent (Invitrogen; Thermo Fisher Scientific, USA). DNA libraries were prepared with Oligo beads and AMPure XP beads. Libraries were quantified on a Qubit 2.0 fluorometer, diluted to 1.5 ng/µl, and assessed for insert size with an Agilent 2100 Bioanalyzer. Sequencing (150-bp paired-end) was performed on an Illumina platform. The reference genome index was built with HISAT2 (v2.0.5), and clean paired-end reads were aligned to the reference genome. Gene-level read counts were obtained using HTSeq (v0.6.0). FPKM values were calculated from gene length and mapped read counts.

### Branched-chain amino acid (BCAA) assay

BCAA levels in cells and tissues were measured using a commercial assay kit (ab83374; Abcam, Cambridge, UK), following the manufacturer’s instructions. Standard curves were generated on 96-well plates using a microplate reader, and sample concentrations were calculated from these curves.

### Western blot (WB) analysis

Total protein was extracted with radioimmunoprecipitation assay buffer (Thermo Fisher Scientific, USA) supplemented with protease and phosphatase inhibitors (Thermo Fisher Scientific, USA). Protein concentrations were determined with a bicinchoninic acid (BCA) kit (Thermo Fisher Scientific, USA). Equal amounts of protein were separated by SDS-PAGE and transferred to nitrocellulose membranes. Membranes were blocked with 5% skim milk for 1 h at room temperature, incubated with primary antibodies overnight at 4 °C, and then with secondary antibodies for 1 h at room temperature. Bands were visualized on an Amersham Imager 600 (GE HealthCare Technologies, Chicago, IL, USA). Primary antibodies are listed in Supplementary Table 1.

### Reverse transcription-quantitative PCR (RT-qPCR)

Total RNA was isolated with TRIzol (Invitrogen, USA) and reverse-transcribed using the PrimeScript RT Reagent Kit (Takara Biotechnology Co., Ltd., Japan) according to the manufacturer’s protocol. qPCR was performed with SYBR Premix Ex Taq (Takara Biotechnology Co., Ltd.) to quantify gene expression. ACTB (β-actin) served as the internal control. Relative expression was calculated using the 2−∆∆Cq method. Primer sequences are provided in Supplementary Table 2.

### Cell transfection

For stable expression, lentiviral plasmids were packaged in HEK293T cells using the GM easy™ Lentiviral Packaging Kit (Genomeditech, Shanghai, China). After transfection, the medium was refreshed at 8 h, and viral supernatants were collected at 48–72 h. Target cells were infected with virus-containing medium supplemented with polybrene (Genomeditech, China) for 24 h, then selected with puromycin (1–2 µg/mL; Genomeditech, China) for 2 weeks. For transient transfection, siRNA and plasmids were introduced into PCa cells using Lipofectamine (Invitrogen, USA). Sequences for all shRNA, siRNA, and plasmids are provided in Supplementary Table 3.

### Cell apoptosis assay

Apoptosis was quantified using the Annexin V–FITC/PI kit (Vazyme, Nanjing, China). Briefly, treated cells were trypsinized (Gibco, USA), washed with PBS, resuspended in binding buffer, stained according to the manufacturer’s instructions, and analyzed by flow cytometry (BD FACSCanto II; BD Biosciences, Franklin, NJ, USA).

### Patient-derived organoid (PDO) culture

PCa PDOs were established and maintained using the Human Prostate Cancer Organoid Culture Medium Kit (Absin, Shanghai, China). Fresh tissues were minced (~ 1–3 mm³) and digested at 37 °C in human PCa primary tissue digestion solution for 10–20 min with gentle agitation. Digestion was quenched by adding primary culture buffer upon microscopic confirmation of abundant single cells or clusters < 70 μm. The suspension was filtered through a 100 μm sieve, and the filtrate was collected. After centrifugation at 300 × g for 5 min, the supernatant was removed, and the pellet was recentrifuged. Cells were resuspended in Matrigel (Corning, NY, USA) at ~ 25× the original tissue volume. Aliquots (25 µL) of the tissue–Matrigel mixture were plated per well of a 24-well plate and solidified at 37 °C for 10–15 min, followed by the addition of organoid culture medium. The medium was changed every 3 days; PDOs were passaged every 1–2 weeks and monitored morphologically.

For drug testing, organoids were collected the day prior and seeded into 96-well plates. Before seeding, 50 µL of 50% Matrigel was added to each well as a base layer. Next, 10 µL of 10% Matrigel containing organoids was added as an intermediate layer, followed by 200 µL of organoid culture medium; plates were incubated for 24 h before treatment.

### Tumor formation assay in nude mice

Male BALB/c nude mice (4–6 weeks; Beijing Vital River Laboratory Animal Technology, Beijing, China) were inoculated subcutaneously in the left axilla with 2 × 10⁶ treated cells. When tumors reached ~ 100 mm³, mice received the indicated treatments (low-BCAA diet, oral olaparib, or oral BCAT2 inhibitor) for one month. Tumor size was measured at predefined time points using the formula: Volume (mm^3^) = 0.5 × width^2^ × length. At the endpoint, mice were euthanized by asphyxiation with CO_2_, and tumors were excised, photographed, and weighed. All animal experiments were conducted following protocols approved by the Animal Ethics Committee of Shanghai Tenth People’s Hospital.

### Nucleoprotein extraction assay

Treated cells (1–10 × 10⁶) were processed for nuclear and cytoplasmic fractionation using the Subcellular Protein Fractionation Kit (Thermo Fisher Scientific, USA) according to the manufacturer’s instructions. The chromatin-bound protein fraction was collected for WB analysis.

### RNA immunoprecipitation (RIP)

RIP was performed with an RNA-binding protein (RBP) immunoprecipitation kit (Absin, China) per the manufacturer’s protocol. Approximately 2 × 10⁷ cells were lysed in RIP lysis buffer on ice for 30 min and clarified at 12,000 × g for 10 min, and the supernatant was collected, with one-quarter of the lysate reserved as input. Protein A/G magnetic beads (Pierce; Thermo Fisher Scientific, USA) were pre-incubated with IgG or target antibodies for 2 h at room temperature, then combined with RIP buffer and cell lysate and incubated overnight at 4 °C. After three washes in RIP buffer, bound RNA was extracted with TRIzol (Invitrogen, USA) and reverse-transcribed using the PrimeScript RT Reagent kit (Takara Biotechnology Co., Ltd., Japan). Immunoprecipitated RNA was quantified by RT-qPCR and RIP-seq. Enrichment of BCAT2 associated with PARP1 was normalized to input BCAT2.

### RIP-seq

rRNA was depleted from immunoprecipitated and input RNA using the Ribo-Zero™ rRNA Removal Kit (Illumina, San Diego, CA, USA). Strand-specific total RNA libraries were prepared with the TruSeq Stranded Total RNA Library Prep Kit (Illumina, USA). Library quality and concentration were assessed on a BioAnalyzer 2100 (Agilent Technologies), and 150-bp paired-end sequencing was performed on an Illumina HiSeq platform.

### Protein-nucleic acid docking assay

The human PARP1 X-ray structure (PDB ID: 5XSR; UniProt ID P09874) was used as the receptor. A partial BCAT2 RNA fragment identified by RIP-seq served as the ligand. RNA structure was predicted with AlphaFold3, and protein–RNA docking was performed using the HDOCK server (http://hdock.phys.hust.edu.cn/). The top 10 poses were ranked by docking score, confidence score, and ligand RMSD; the highest-scoring model was selected as the optimal complex. Models were visualized in PyMOL 2.4 (https://pymol.org/).

### RNA pull-down

BCAT2 mRNA was transcribed in vitro using the MAXIscript™ T7 Transcription Kit (Thermo Fisher Scientific, USA) and 3′-end labeled by desthiobiotinylation (Pierce™ RNA 3′ End Desthiobiotinylation Kit; Thermo Fisher Scientific, USA). Biotinylated RNA was incubated with streptavidin magnetic beads (Thermo Fisher Scientific, USA) for 2 h at room temperature. Cell lysates were prepared with Pierce IP Lysis Buffer (Thermo Fisher Scientific, USA) and incubated with the biotin-labeled BCAT2 RNA at 4 °C for 1 h. Bound RNA–protein complexes were eluted with Biotin Elution Buffer (Thermo Fisher Scientific, USA) according to the Pierce Magnetic RNA–Protein Pull-Down Kit instructions. Proteins captured with sense or antisense BCAT2 RNA were identified, and eluates were analyzed by Western blot. Primer sequences used for PCR are listed in Supplementary Table 2.

### Lactate assay

L-lactate was quantified using an L-Lactate Assay Kit (ab65331; Abcam, UK) per the manufacturer’s protocol. Standard curves were generated on 96-well plates using a microplate reader, and sample concentrations were calculated from these curves.

### Seahorse assay

Cell proliferation and glycolysis stress assays were conducted using a Seahorse XF103015 instrument (Agilent, CA, USA) according to the manufacturer’s protocol. Cells were seeded one day prior to measurement at 1 × 10⁶ cells/well with ≥ 6 technical replicates per condition. For the mitochondrial stress test, oligomycin (2 µM), FCCP (1 µM), and rotenone/antimycin A (0.5 µM) were injected to assess the OCR. For the glycolysis stress test, glucose (10 mM), oligomycin (1 µM), and 2-deoxy-D-glucose (2-DG; 50 mM) were injected to determine the ECAR.

### CUT&Tag assay

Cells were harvested and incubated with pre-washed ConA beads for 10 min at room temperature. After washes, samples were incubated with primary antibody against H3K18la (PTM-1427RM) for 2 h at room temperature, followed by a 30-min incubation with secondary antibody. PA-Tn5 transposome (1.2 µL) was added and incubated for 30 min at room temperature. Beads were resuspended in 30 µl of 10 mM MgCl₂ and incubated at 37 °C for 1 h to initiate tagmentation. DNA was extracted and purified, and libraries were amplified and purified with XP beads (Beckman Coulter, FL, USA). Library size distribution was assessed on an Agilent 4200 TapeStation, libraries were pooled to the desired proportions, and final concentrations were adjusted per the manufacturer’s recommendations. Sequencing was performed on an Illumina NovaSeq 6000 platform (150-bp paired-end).

### Chromatin immunoprecipitation (CHIP) assay

ChIP was conducted using a commercial kit (Absin, China) following the manufacturer’s instructions. PCa cells were cross-linked with 1% formaldehyde, lysed, and sonicated to yield chromatin fragments averaging 500–1,000 bp. Immunoprecipitation was carried out with the indicated antibodies. DNA was purified and quantified via RT-qPCR. Enrichment was calculated as a percentage of input DNA. Primer sequences for ChIP and RT-qPCR are listed in Supplementary Table 2.

### Immunohistochemical (IHC) analysis

A total of 516 patients with PCa who underwent radical prostatectomy were enrolled. Use of clinical specimens was approved by the Ethics Committee of Shanghai East Hospital, Tongji University, and informed consent was obtained from all participants. After deparaffinization and ethanol dehydration, tissue microarrays underwent heat-induced antigen retrieval. Sections were incubated overnight at 4 °C with primary antibodies, followed by incubation with species-appropriate secondary antibodies. After chromogenic development, dehydration, and mounting, slides were evaluated by experienced pathologists according to WHO histological criteria for PCa. Staining intensity was scored as 0 (negative), 1 (weak), 2 (moderate), or 3 (strong); the proportion of positive cells as 1 (0–25%), 2 (26–50%), 3 (51–75%), or 4 (76–100%). The final IHC score equaled intensity × proportion [32].

### Statistical analysis

Statistical analyses were performed using R 4.1.1 and Prism 9.0 (GraphPad Software, LLC, San Diego, CA, USA). Group differences were assessed using two-tailed Student’s t-tests or one-way ANOVA, as appropriate. Associations between categorical variables were evaluated using the chi-square test. Image quantification was carried out with ImageJ software (https://imagej.net/ij/), and flow cytometry data were analyzed with FlowJo V10 (https://www.flowjo.com/). Quantified results are presented as the means ± SD. A p-value < 0.05 was considered statistically significant, with the following notation used: *p < 0.05, **p < 0.01, ***p < 0.001; and ns, not significant.

## Results

### BCAAs and BCAT2 are associated with PARPi resistance in PCa

**Fig. 1:**
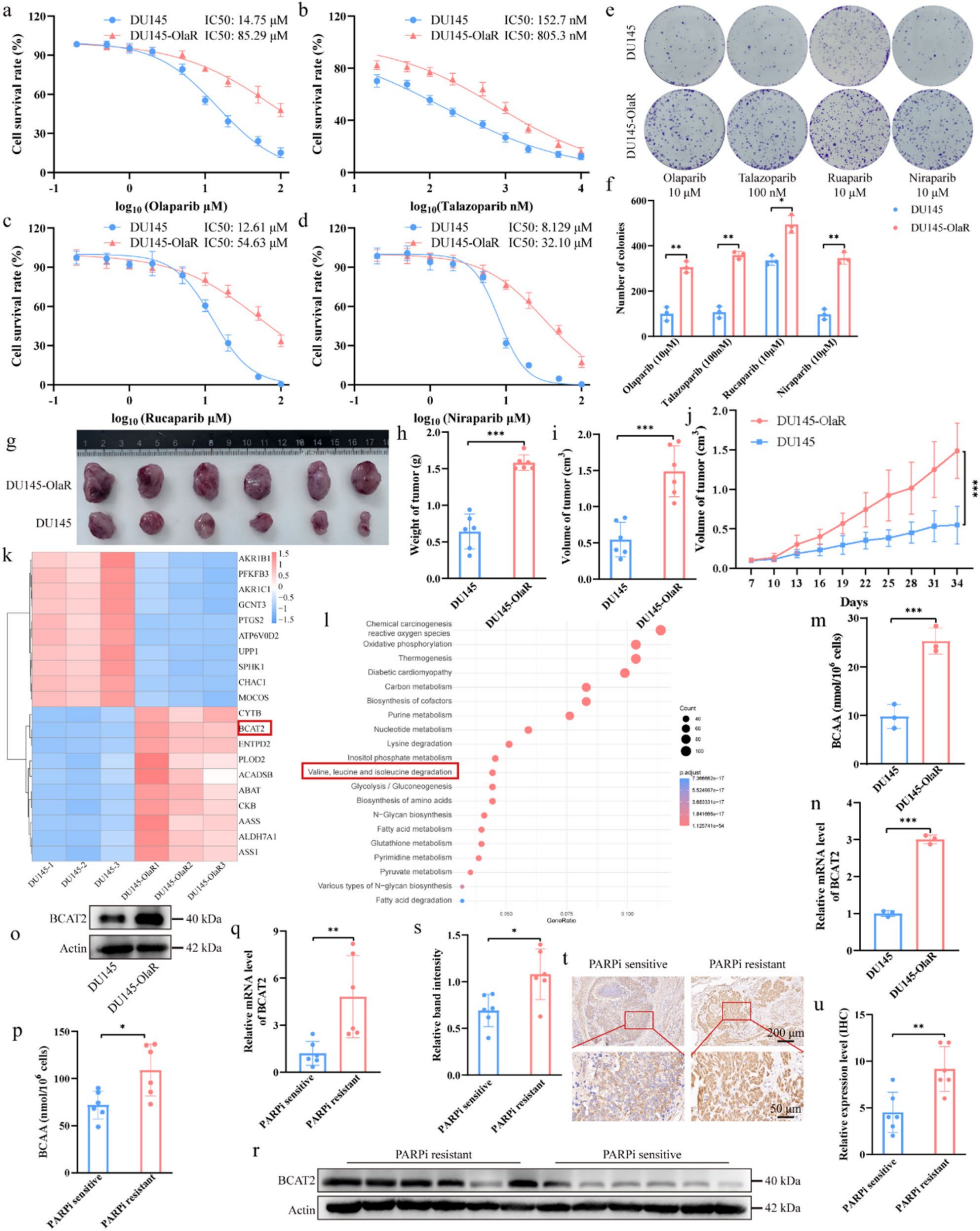
Upregulated BCAT2 expression correlates with PARPi resistance in PCa.** a** olaparib, **(b)** talazoparib, **(c)** rucaparib and **(d)** niraparib IC_50_ curves of wild-type DU145 and cells with acquired resistance to olaparib. **e**,** f** Clonogenic assays of DU145 and DU145-OlaR cells in the presence of PARPi for 10–14 days (olaparib: 10µM; talazoparib: 100 nM; rucaparib: 10 µM; niraparib: 10 µM). **g** Sensitivity of DU145 and DU145-OlaR cells to olaparib in nude mice. **h** Tumor weight, **(i)** volume, and **(j)** growth curves were statistically analyzed. **k** Heatmap illustrating differentially expressed metabolism-related genes between DU145 and DU145-OlaR groups by RNA sequencing. **l** The top 20 enriched KEGG metabolic pathways in DU145-OlaR with DU145 cells. **m** Analysis of BCAA levels in DU145 and DU145-OlaR cells. **n** BCAT2 mRNA levels analyzed by RT-qPCR in DU145-OlaR and DU145 cells. **o** Western blot analysis of BCAT2 protein levels. **p** BCAA level analysis in PARPi-resistant (*n* = 6) and sensitive (*n* = 6) PCa fresh-frozen tissues. **q** BCAT2 mRNA levels in PARPi-resistant and sensitive PCa tissues. **r**,** s** WB analysis of BCAT2 protein levels in PARPi-resistant and sensitive tissues. **t** IHC staining and **(u)** quantification analysis of BCAT2 expression in PARPi-resistant and sensitive PCa tissues

Olaparib-resistant DU145 cells (DU145-OlaR) were generated to investigate metabolic mechanisms underlying PARPi resistance in PCa. The half-maximal inhibitory concentration (IC₅₀) for DU145-OlaR was 82.29 µM versus 14.75 µM in the wild-type DU145 cells (Fig. 1a). To assess the breadth of PARPi resistance, we determined the IC_50_ values of DU145-OlaR cells against additional clinically relevant PARP inhibitors for PCa. The cells exhibited broad cross-resistance, with markedly increased IC_50_ values for Talazoparib, Rucaparib, and Niraparib (Fig. 1b-d). Consistently, PARPi treatment markedly reduced clonogenicity in DU145 but not in DU145-OlaR under identical conditions (Fig. 1e-f). The olaparib-resistant phenotype of DU145-OlaR cells was further confirmed in a mouse xenograft model. DU145-OlaR-derived tumors exhibited markedly enhanced growth relative to parental DU145 tumors upon olaparib treatment (50 mg/kg, 3 times/week for 3 weeks), as evidenced by significantly increased tumor volume and weight at the endpoint (Fig. 1g-j).

RNA-seq profiling revealed strong intra-group homogeneity and clear inter-group separation between DU145 and DU145-OlaR (Supplementary Fig. 1a-b). Intersecting differentially expressed genes with Kyoto Encyclopedia of Genes and Genomes (KEGG) metabolic regulators yielded 883 candidates (Supplementary Fig. 1c). BCAT2 emerged as conspicuously upregulated (Fig. 1k, Supplementary Fig. 1d), and KEGG analysis indicated dysregulation of valine, leucine, and isoleucine degradation (Fig. 1l). Given prior links between BCAT2 and PCa progression [27] and its unexplored role in PARPi resistance, we prioritized BCAT2 for further study.

To validate these findings, we quantified BCAAs and assessed BCAT2 expression by RT-qPCR and WB in DU145 and DU145-OlaR. DU145-OlaR displayed elevated BCAAs and increased BCAT2 at mRNA and protein levels (Fig. 1m-o). BCAT2 was also consistently upregulated at transcriptional and translational levels following PARPi exposure (Supplementary Fig. 1e-j). In a clinical cohort of six paired PARPi-sensitive and -resistant cases, resistant tumors had significantly higher BCAA levels and BCAT2 expression (Fig. 1p-s). Immunohistochemical (IHC) analysis in PARPi-resistant versus -sensitive PCa tissues confirmed BCAT2 upregulation in resistant groups (Fig. 1t-u).

### BCAT2-regulated BCAA metabolism regulates PARPi sensitivity in PCa

**Fig. 2:**
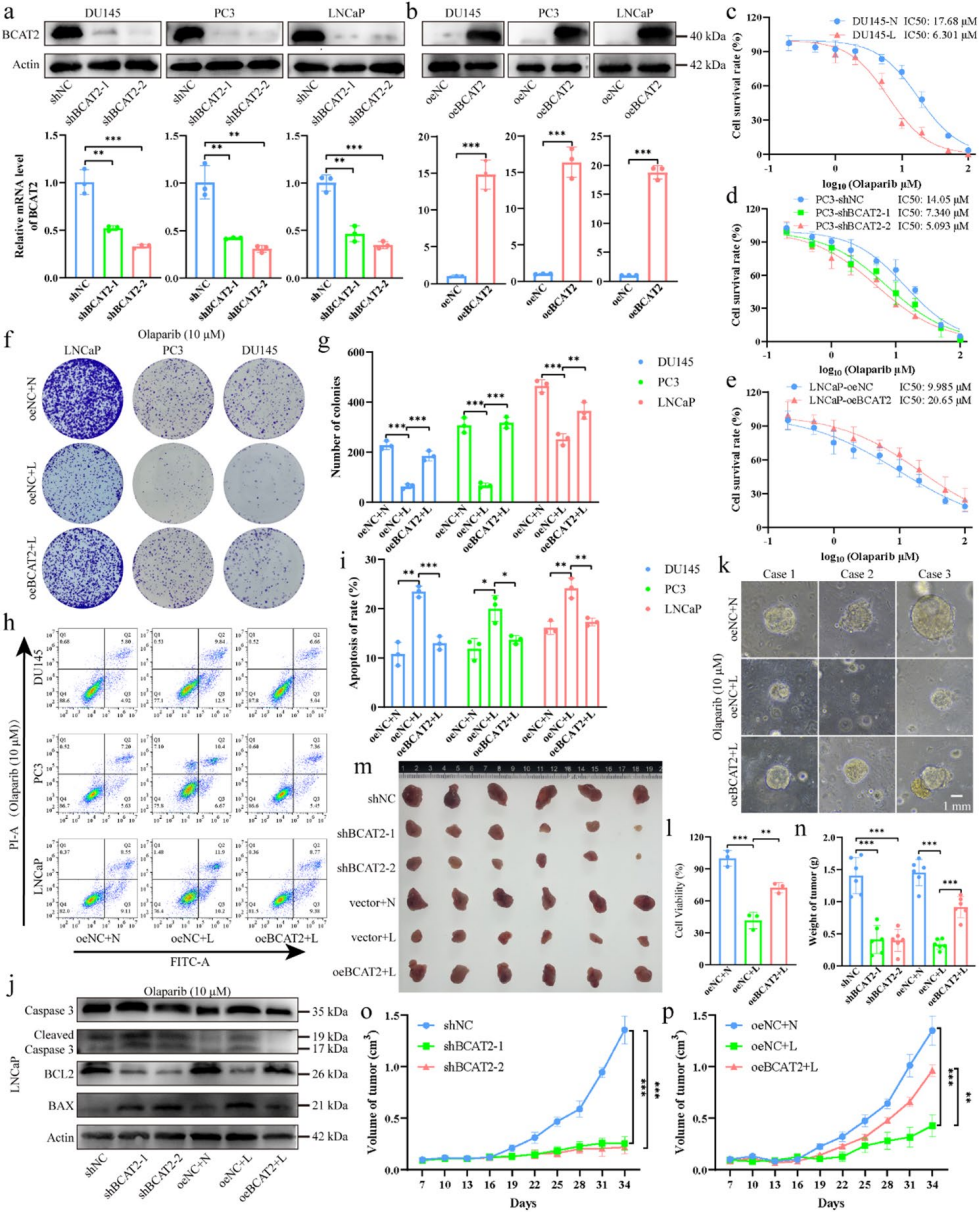
BCAT2 regulates olaparib sensitivity and promotes PARPi resistance in PCa.** a** BCAT2 knockdown significantly reduced its expression in PCa cells. **b** BCAT2 overexpression significantly increased its expression in PCa cells. **c** Olaparib IC_50_ curves in DU145 cells under low BCAA (-L) vs. normal (-N) culture conditions. **d** Olaparib IC_50_ curves in shBCAT2 PC3 cells. **e** Olaparib IC_50_ curves in oeBCAT2 LNCaP cells. **f**,** g** Clonogenic assays of PCa cells in the presence of PARPi for 10–14 days (Olaparib: 10 µM). **h**,** i** Apoptotic rate of PCa cells treated with olaparib (10 µM) for 48 h. **j** WB analysis of apoptosis-related protein levels in LNCaP cells, treated with olaparib (10 µM) for 48 h. **k**,** l** The sensitivity of PDOs to olaparib. Organoids were treated with 10 µM olaparib for 5 days. **m** Sensitivity of DU145 cells to olaparib in nude mice. **n** Tumor weight and **(o**,** p)** volume growth curves were statistically analyzed

To investigate the role of BCAT2 and BCAAs in PARPi resistance, we evaluated their impact on olaparib sensitivity in PCa. To ensure the consistency and reproducibility of the results, we used the same validated shRNA sequences that were employed in the previous study. Stable BCAT2 knockdown (shBCAT2) and overexpression (oeBCAT2) lines were generated in DU145, PC3, and LNCaP cells (Fig. 2a-b). Differences in knockdown efficiency between cell lines reflect inherent variations in lentiviral transduction efficiency, proliferation rates, and basal BCAT2 expression levels. We then assessed olaparib sensitivity using IC_50_ and colony-formation assays. BCAT2 knockdown or reduced BCAA levels (1/5 the concentration in standard medium) significantly increased olaparib sensitivity, whereas BCAT2 overexpression promoted cell viability under olaparib treatment (Fig. 2c-e, Supplementary Fig. 2a-c). Furthermore, we found that compared with DU145 and PC3 cells, LNCaP cells exhibited a lower initial resistance to olaparib. This result might be attributed to the AR signaling pathway, BRCA/HR status, the different metabolic phenotypes of AR-negative cells, and even different cell states. BCAT2 knockdown also suppressed clonogenic ability following olaparib treatment (Supplementary Fig. 2d-e). Reduced BCAA levels decreased clonogenic capacity in response to olaparib, an effect reversed by BCAT2 overexpression (Fig. 2f-g). Flow-cytometric apoptosis assays further indicated that BCAT2 knockdown or BCAA reduction enhanced olaparib sensitivity (Fig. 2h-i, Supplementary Fig. 2f-g). Moreover, BCAT2 overexpression reversed the increased sensitivity induced by low BCAA levels (Fig. 2h-i). Western blotting confirmed corresponding changes in apoptosis-related protein expression (Fig. 2j, Supplementary Fig. 2h).

Consistent with these findings, Patient-derived organoid (PDO) models showed that BCAT2 and BCAAs modulate olaparib responses in PCa (Fig. 2k-l, Supplementary Fig. 2i-j). We next assessed whether BCAT2 altered the tumor response to olaparib in a xenograft mouse model. Nude mice were subcutaneously injected with treated DU145 cells. When tumors reached ~ 100 mm³, mice were administered 50 mg/kg olaparib by oral gavage or fed a low-BCAA diet for one month. Both BCAT2 knockdown and BCAA restriction markedly suppressed tumor growth and weight, whereas BCAT2 overexpression reversed the antitumor effect conferred by low BCAA levels (Fig. 2m-p). These results indicate that BCAT2-regulated BCAA metabolism modulates olaparib sensitivity and underlies PARPi resistance in PCa, both in vitro and in vivo.

### RNA-binding activity of PARP1 regulates BCAT2 expression

**Fig. 3:**
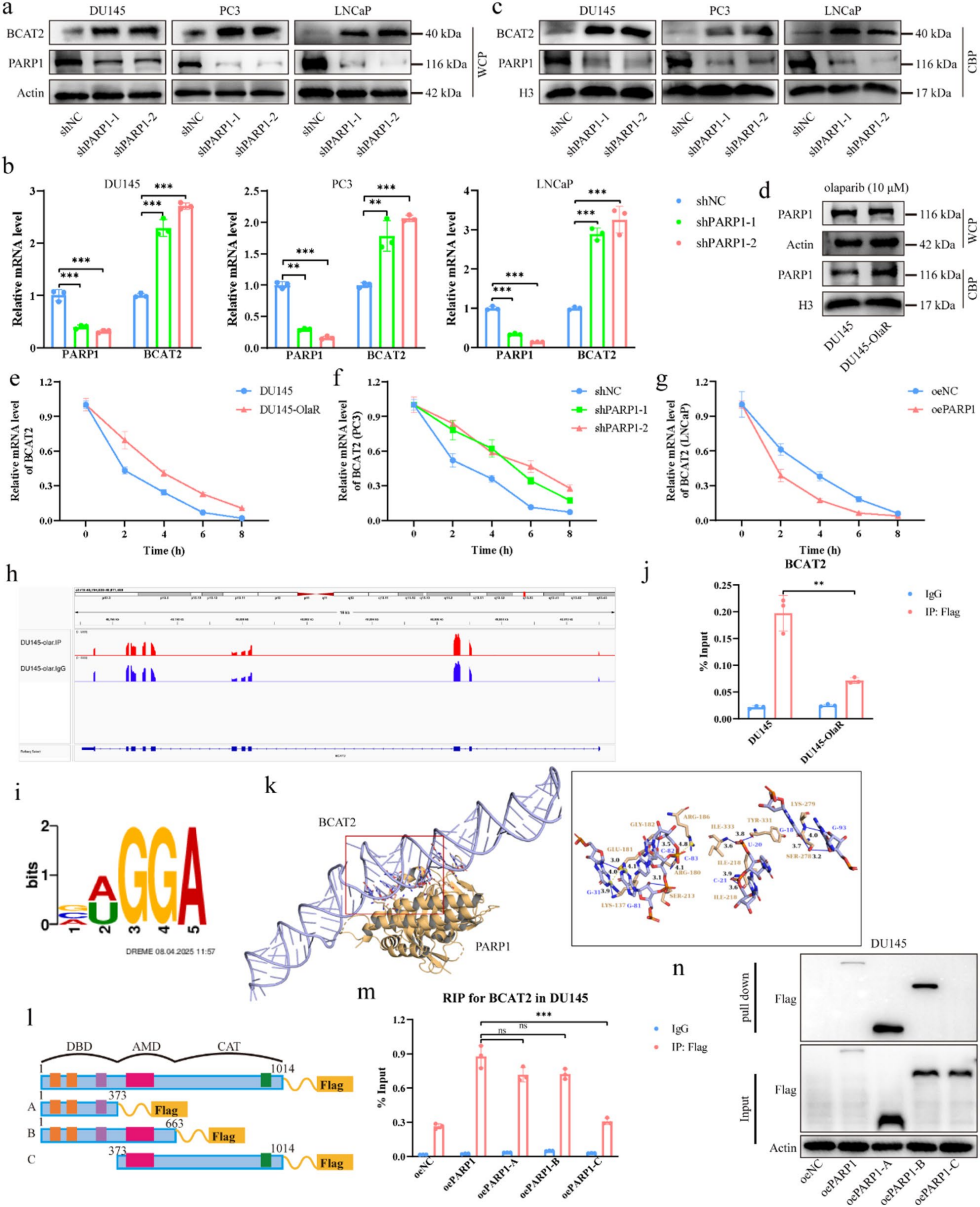
The binding of PARP1 protein and BCAT2 mRNA influences BCAT2 expression.** a** Western blot analysis of the whole-cell protein (WCP) in PARP1 knockdown PCa cells. **b** RT-qPCR analysis of the BCAT2 and PARP1 mRNA levels in PARP1 knockdown PCa cells. **c** Western blot analysis of the chromatin-binding protein (CBP) in PARP1 knockdown PCa cells. **d** PARP1 protein expression levels of WCP or CBP in DU145 and DU145-OlaR cells treated with 10 µM olaparib for 48 h. **e-g** The BCAT2 mRNA level was detected in PCa cells. The cells were treated with 4 µM actinomycin D (ACTD, MedChemExpress, USA) for a specified time. **h** IGV tracks showing the enrichment of PARP1 in BCAT2 gene regions in DU145-OlaR cells. **i** A GA-rich motif enriched in the site PARP1 specifically binding to BCAT2 mRNA. **j** RIP-qPCR was used to examine RNA enrichment in DU145 and DU145-OlaR cells. **k** Computational docking analysis of PARP1 protein and BCAT2 mRNA fragment identified by RIP-seq. **l** Schematic diagram of the full-length PARP1 or various truncated mutants. **m** RIP-qPCR analysis in DU145 cells transfected with various truncated mutants. **n** RNA–protein pull-down assays in DU145 cells transfected with various truncated mutants

To investigate the mechanism of PARPi-induced BCAT2 upregulation, we prepared chromatin-binding protein (CBP) and whole-cell protein (WCP) fractions and performed WB analysis. PARP1 knockdown (shPARP1) in PCa cells increased BCAT2 protein and mRNA expression (Fig. 3a-c), whereas PARP1 overexpression (oePARP1) significantly reduced both (Supplementary Fig. 3a-c). We found that the expression levels of BCAT2 in DU145 cells and LNCaP cells were different, which might be attributed to inherent changes in basal transcription, mRNA stability, and transfection efficiency. We next assessed CBP and WCP levels of PARP1 in DU145 and DU145-OlaR cells treated with 10 µM olaparib for 48 h. PARP1 CBP was increased in DU145-OlaR cells, whereas PARP1 WCP and mRNA levels were largely unchanged. These findings suggest that PARP1 DNA trapping induced by PARP inhibitors elevates PARP1 in the chromatin-bound fraction and is associated with increased BCAT2 expression (Fig. 3d, Supplementary Fig. 3d). PARP1 did not affect the half-life or protein stability of BCAT2 (Supplementary Fig. 3e-g), and PARP1 expression was not influenced by BCAT2 (Supplementary Fig. 3h-i).

Based on these results and the RBP function of PARP1, we hypothesized that enhanced chromatin binding reduces the free PARP1 available for RNA binding, thereby weakening PARP1 binding to BCAT2 mRNA, slowing mRNA decay, and upregulating BCAT2 expression. To assess BCAT2 mRNA stability, we treated cells with 4 µM actinomycin D (ACTD; MedChemExpress, USA) to halt transcription and monitored BCAT2 levels by RT-qPCR over time. BCAT2 mRNA decay was slower in DU145-OlaR and shPARP1 cells, whereas PARP1 overexpression accelerated its degradation (Fig. 3e-f, Supplementary Fig. 4a-d). To verify PARP1’s RBP function, we performed RNA immunoprecipitation sequencing (RIP-seq); PARP1 specifically bound BCAT2 mRNA at a site containing a GA-rich motif, a classic RBP-binding feature (Supplementary Fig. 4e, Fig. 3h-i). KEGG enrichment analysis indicated that PARP1-bound RNAs were closely associated with BCAA metabolic pathways (Supplementary Fig. 4f).

To validate this interaction, RIP-qPCR showed that PARP1 binding to BCAT2 mRNA was significantly weakened in DU145-OlaR cells compared with DU145 cells (Fig. 3j), whereas the binding increased when BCAT2 was overexpressed (Supplementary Fig. 4g). Computational docking further supported the interaction, revealing favorable binding between the PARP1 DNA-binding domain (DBD) and the BCAT2 mRNA fragment identified by RIP-seq (Fig. 3k). To determine the PARP1 region responsible for BCAT2 interaction, we performed RIP using full-length PARP1 and truncated mutants (Fig. 3l). The results indicated that BCAT2 mainly interacts with the DBD domain of PARP1 (Fig. 3m, Supplementary Fig. 4h-i). As a reciprocal approach, RNA–protein pull-down assays confirmed specific binding between BCAT2 mRNA and PARP1; this binding was attenuated in DU145-OlaR cells and enhanced in PARP1-overexpressing cells (Supplementary Fig. 4j-k). Binding localized primarily to the PARP1 DBD, consistent with the RIP results (Fig. 3n, Supplementary Fig. 4l).

To determine whether BCAT2 inhibition could increase endogenous DNA damage, we examined γ-H2AX levels. The results of the WB experiment show that BCAT2 knockdown significantly increased γ-H2AX protein levels, indicating elevated endogenous DNA damage (Supplementary Fig. 5a). Furthermore, combination treatment with olaparib further enhanced γ-H2AX accumulation compared to single treatments, supporting that BCAT2 reduction increases PARP1 trapping by elevating DNA damage burden (Supplementary Fig. 5a). To determine whether USP1 stabilizes BCAT2 through deubiquitination, we examined BCAT2 protein and ubiquitination levels following USP1 inhibition. Treatment with the USP1-specific inhibitor ML323 (10 µM, 6 h, MedChemExpress) markedly reduced BCAT2 protein abundance (Supplementary Fig. 5b), accompanied by a significant increase in its ubiquitination (Supplementary Fig. 5c). Moreover, co-treatment with the proteasome inhibitor MG132 (10 µM, 4 h, MedChemExpress) reversed the ML323-induced decrease in BCAT2 protein levels (Supplementary Fig. 5d). Together, these results demonstrate that USP1 stabilizes BCAT2 by promoting its deubiquitination.

### BCAT2 promotes PARPi resistance through H3K18la lactylation in PCa

**Fig. 4:**
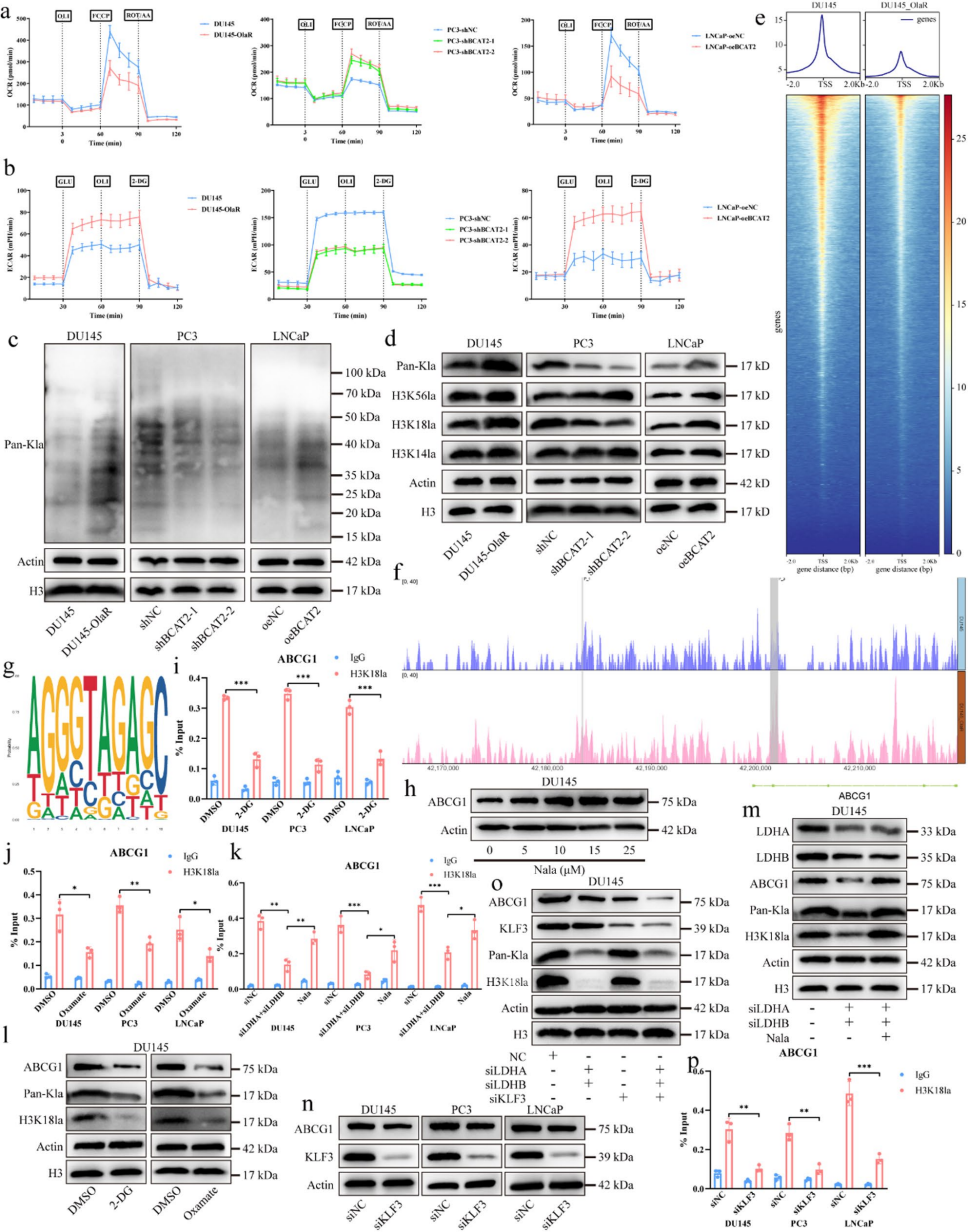
BCAT2 regulates ABCG1 expression via H3K18la lactylation.** a** Dynamic oxygen consumption rate (OCR) (*n* = 6) measurements in PCa cells. **b** Extracellular acidification rate (*n* = 6) measurements in PCa cells. **(c)** Western blotting analysis of global lactylation levels in PCa cells. **d** Western blotting analysis of some histone lactylation sites’ levels in PCa cells. **e** Heatmaps of H3K18la levels detected by CUT&Tag around gene body regions in DU145 and DU145-OlaR cells. **f** IGV tracks showing the enrichment of H3K18la in ABCG1 gene regions in DU145 and DU145-OlaR cells. **g** The canonical DNA-binding motif of transcription factor KLF3. **h** Western blotting analysis of ABCG1 level in DU145 cells treated with different concentrations of sodium lactate (Nala) for 48 h. **i-k** ChIP–qPCR assay of H3K18la status in the ABCG1 genomic region in PCa cells upon treatment with histone lactylation inhibitors (2-DG or Oxamate) or LDHA/B inhibition. **l** Western blot analysis of ABCG1, Pan-Kla, and H3K18la levels in DU145 cells upon histone lactylation inhibitors (2-DG or Oxamate). **m** Western blot analysis of ABCG1, Pan-Kla, and H3K18la levels in DU145 cells upon LDHA/B inhibition. **n** Western blot analysis in KLF3 knockdown PCa cells. **o** Western blot analysis of ABCG1 levels in DU145 cells upon LDHA/B inhibition or KLF3 knockdown. **p** ChIP–qPCR assay of H3K18la status in the ABCG1 genomic region in PCa cells upon knockdown of KLF3

RNA-seq analysis revealed that PARPi resistance is associated with metabolic pathways such as glycolysis and oxidative phosphorylation, both closely linked to lactate metabolism (Fig. 1l). Within the BCAT2-mediated “valine, leucine, and isoleucine degradation” pathway, BCAA catabolites can enter the TCA cycle and ultimately be converted to lactate. We therefore hypothesized that BCAT2 drives PARPi resistance by regulating lactate production and downstream lactylation. Compared with control cells, DU145-OlaR and BCAT2-overexpressing PCa cells exhibited significantly elevated lactate levels, which were suppressed by BCAT2 knockdown (Supplementary Fig. 6a-b). Seahorse analysis showed a pronounced Warburg effect in DU145-OlaR and BCAT2-overexpressing cells—decreased mitochondrial respiration (oxygen consumption rate [OCR]) and increased glycolysis (extracellular acidification rate [ECAR])—which was attenuated in shBCAT2 cells (Fig. 4a-b). Lactate accumulation was accompanied by elevated global lactylation in DU145-OlaR cells. BCAT2 modulated this effect: overexpression increased and knockdown decreased lactylation levels (Fig. 4c, Supplementary Fig. 6c). Among histone lactylation marks, only H3K18la was significantly associated with PARPi resistance and BCAT2, showing increased levels in PARPi-resistant PCa cells (Fig. 4d, Supplementary Fig. 6d). Differential lactylation levels among cell lines reflect their distinct metabolic phenotypes, AR status, and basal glycolytic rates. We attribute the observed differences in H3K18la and lactylation levels among different cells primarily to their distinct intrinsic metabolic phenotypes and the regulatory influence of AR signaling. Specifically, the AR-negative DU145 and PC3 lines, which model CRPC, display a markedly enhanced glycolytic flux and elevated basal lactate production compared to the AR-positive LNCaP cells. This Warburg-like metabolic rewiring in the AR-negative models increases global lactate availability, thereby promoting histone lactylation. Furthermore, active AR signaling in LNCaP cells directly suppresses glycolysis and promotes oxidative phosphorylation through the regulation of metabolic genes, which consequently reduces lactate generation and downstream lactylation levels, consistently with their AR-negative counterparts.

To systematically investigate mechanisms of BCAT2-mediated PARPi resistance, we performed H3K18la CUT&Tag in DU145 and DU145-OlaR cells to profile genome-wide distributions. We analyzed H3K18la enrichment within ± 3 kb of gene bodies and profiled genome-wide occupancy across both cell states (Fig. 4e). The genomic distribution of CUT&Tag signals supported H3K18la as a transcription-associated mark (Supplementary Fig. 6e). KEGG analysis of CUT&Tag-derived differentially expressed genes in DU145-OlaR cells showed significant enrichment of the “ABC transporters” pathway, with ABCG1 displaying the strongest signal (Supplementary Fig. 6f). Integrative Genomics Viewer (IGV) revealed H3K18la enrichment at the ABCG1 promoter (Fig. 4f). The canonical DNA-binding motif of the transcription factor KLF3 was identified within the H3K18la-marked region at the ABCG1 promoter (Fig. 4g). We next assessed whether lactate influences ABCG1 expression. Western blotting demonstrated a dose-dependent increase in ABCG1 levels with rising lactate concentrations (Fig. 4h, Supplementary Fig. 6g). H3K18la chromatin immunoprecipitation (ChIP) assays demonstrated that both histone lactylation inhibitors (2-DG and Oxamate) and LDHA/B inhibition resulted in significantly reduced H3K18la enrichment at the ABCG1 promoter (Fig. 4i-k), accompanied by decreased ABCG1 RNA and protein levels (Fig. 4l-m, Supplementary Fig. 6h-l). To evaluate the function of KLF3 in ABCG1 transcription and PARPi resistance, we performed loss- and gain-of-function experiments. KLF3 knockdown (siKLF3) reduced ABCG1 expression, and its overexpression elevated it (Fig. 4n, Supplementary Fig. 6m). Combined KLF3 knockdown and lactylation inhibition further augmented ABCG1 downregulation (Fig. 4o, Supplementary Fig. 6n). ChIP assays demonstrated that KLF3 knockdown substantially reduced H3K18la enrichment at the ABCG1 promoter (Fig. 4p). Taken together, these results indicate that BCAT2 promotes lactate accumulation, which facilitates H3K18la-mediated recruitment of KLF3 to the ABCG1 promoter, thereby activating its transcription and contributing to PARPi resistance in PCa.

Elevated ABCG1 expression has been associated with poor prognosis in multiple cancer types. In prostate cancer specifically, increased ABCG1 levels correlate with enhanced cancer stem cell (CSC) properties, which are known to drive tumor progression, metastasis, and therapeutic resistance. To determine whether the observed increase in CSC characteristics in the BCAT2-overexpressing cell model is linked to upregulation of ABCG1, we assessed the expression of CSC markers, including CD133 and ALDH1. Overexpression of BCAT2 markedly increased the protein levels of the CSC markers CD133 and ALDH1 compared to control cells, paralleling the upregulation of ABCG1 (Supplementary Fig. 7a). Conversely, BCAT2 knockout reduced the expression of these CSC markers (Supplementary Fig. 7b). Together, these results suggest that the increase in ABCG1 levels is related to the regulation of tumor stemness regulated by BCAT2. Furthermore, we also evaluated the effects of BCAT2 inhibition and the combination therapy with olaparib on the expression of ABCG1 and H3K18la. Since PARP inhibitors can mediate an increase in the expression of BCAT2, thereby inducing an increase in the expression of ABCG1, after the use of olaparib monotherapy, the protein level of ABCG1 and H3K18la increase, while they decrease after knocking down BCAT2. After the combined treatment with BCAT2 inhibition and olaparib, the levels of ABCG1 and H3K18la were also significantly suppressed (Supplementary Fig. 7c).

### PARP1-BCAT2-ABCG1 axis regulates PARPi resistance in PCa

**Fig. 5:**
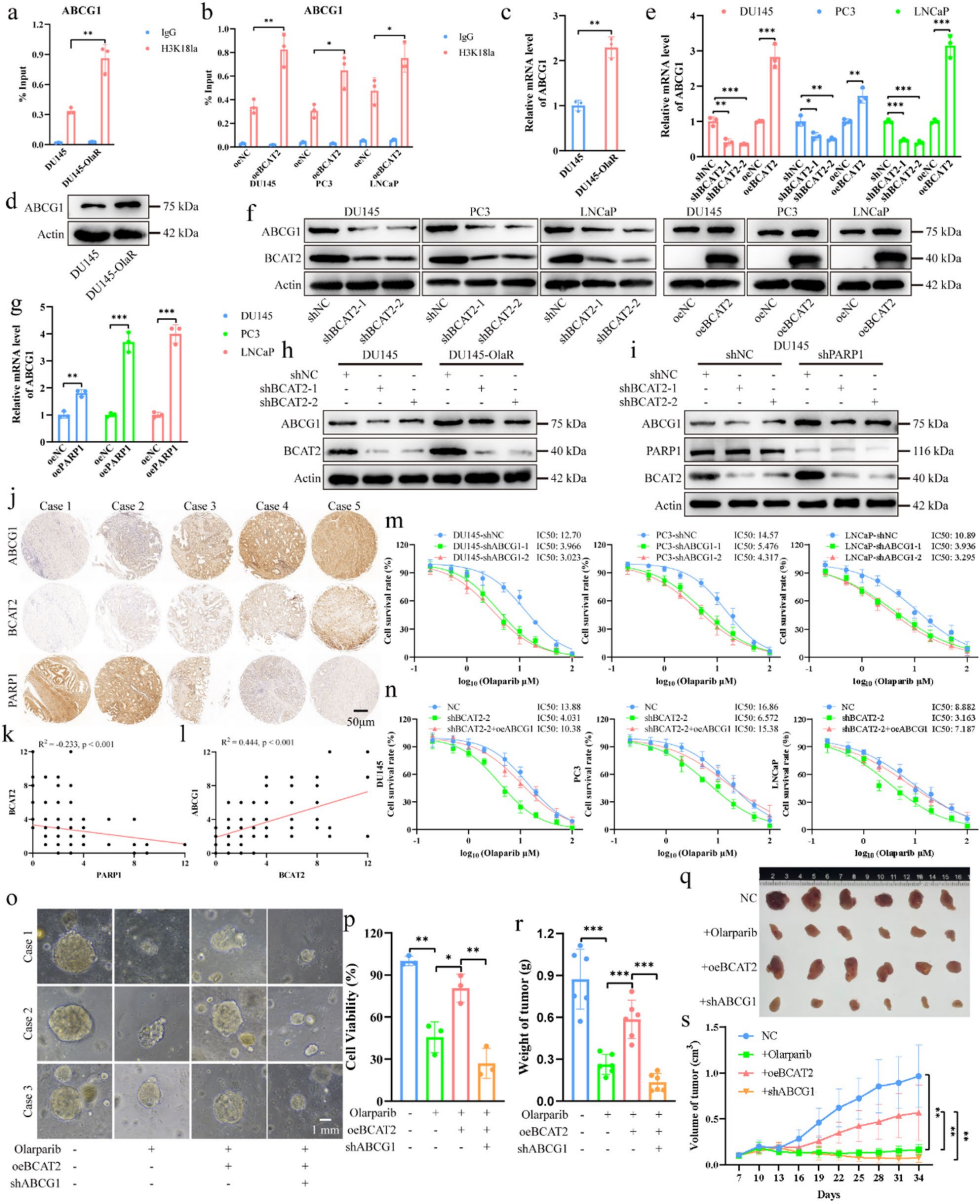
PARP1-BCAT2-ABCG1 axis contributes to PARPi resistance.** a** ChIP–qPCR assay of H3K18la status in the ABCG1 genomic region in DU145 and DU145-OlaR cells. **b** ChIP–qPCR assay of H3K18la status in the ABCG1 genomic region in BCAT2 overexpression cells. **c** RT-qPCR and **(d)** Western blot analysis of ABCG1 level in DU145 and DU145-OlaR cells. **e** RT-qPCR and **(f)** Western blot analysis of ABCG1 expression in BCAT2 knockdown or overexpression cells. **g** RT-qPCR analysis of ABCG1 mRNA levels in PARP1 overexpression cells. **h** Western blotting analysis in DU145 and DU145-OlaR cells, which were transfected with shNC or shBCAT2, respectively. **i** Western blotting analysis in shNC- and shPARP1-DU145 cells, which were transfected with shNC or shBCAT2, respectively. **j** Immunohistochemical staining of PARP1, BCAT2, and ABCG1 in PCa tissue chips. **k** Correlation analysis between PARP1 and BCAT2. **l** Correlation analysis between BCAT2 and ABCG1. **m** Olaparib IC_50_ curves in shABCG1 PCa cells. **n** Olaparib IC_50_ curves in PCa cells treated with shBCAT2 and/or oeABCG1. **o**,** p** The cell viability of PDOs after 5 days of designated treatment. **q** Different groups of DU145 cells were injected subcutaneously into nude mice. **r** Tumor weight and **(s)** volume growth curves were statistically analyzed

As ABCG1 was identified downstream of BCAT2/H3K18la in PCa, we next examined the functional significance of the PARP1–BCAT2–ABCG1 axis in PARPi-resistant cells. ChIP–qPCR in DU145-OlaR cells showed increased H3K18la enrichment at ABCG1 promoter regions compared with DU145 cells (Fig. 5a). With BCAT2 knockdown or overexpression, H3K18la enrichment at the ABCG1 promoter correspondingly decreased or increased (Fig. 5b, Supplementary Fig. 8a-c). Consistently, ABCG1 mRNA and protein levels were elevated in DU145-OlaR cells (Fig. 5c-d). ABCG1 protein and mRNA levels decreased with BCAT2 knockdown and increased with BCAT2 overexpression (Fig. 5e-f), whereas the opposite pattern was observed in shPARP1 or oePARP1 cells (Fig. 5g, i, Supplementary Fig. 8d). To determine whether BCAT2-mediated ABCG1 upregulation is required in DU145-OlaR and shPARP1 cells, we knocked down BCAT2 and measured ABCG1 protein. BCAT2 knockdown reduced ABCG1 expression in both DU145-OlaR and shPARP1 cells (Fig. 5h-i, Supplementary Fig. 8d). By contrast, knockdown or overexpression of ABCG1 (shABCG1 or oeABCG1) did not substantially alter BCAT2 or PARP1 levels (Supplementary Fig. 8e). In PCa tissues, IHC analysis showed a negative correlation between PARP1 and BCAT2 and a positive correlation between BCAT2 and ABCG1 (Fig. 5j-l).

To test whether BCAT2-induced ABCG1 accumulation contributes to PARPi resistance, we knocked down ABCG1 in PCa cells and overexpressed ABCG1 in shBCAT2 cells. ABCG1 knockdown significantly reduced the olaparib IC_50_ (Fig. 5m), whereas ABCG1 overexpression rescued the IC_50_ decrease caused by BCAT2 knockdown (Fig. 5n). ABCG1 knockdown sensitized PCa cells to olaparib, reducing colony formation and increasing apoptosis (Supplementary Fig. 8f-i). Conversely, ABCG1 overexpression reversed the sensitization phenotype induced by BCAT2 knockdown, indicating that ABCG1 mediates BCAT2-conferred PARPi resistance (Supplementary Fig. 8j-m). In PDOs, olaparib markedly suppressed growth, whereas BCAT2 overexpression conferred resistance; ABCG1 knockdown restored olaparib sensitivity in BCAT2-overexpressing PDOs (Fig. 5o-p). Consistent results were obtained in mouse subcutaneous tumor models (Fig. 5q-s), collectively supporting the conclusion that the PARP1-BCAT2-ABCG1 axis regulates PARPi resistance in PCa.

### BCAT2 inhibition synergizes with PARP inhibitors in vitro and in vivo

**Fig. 6:**
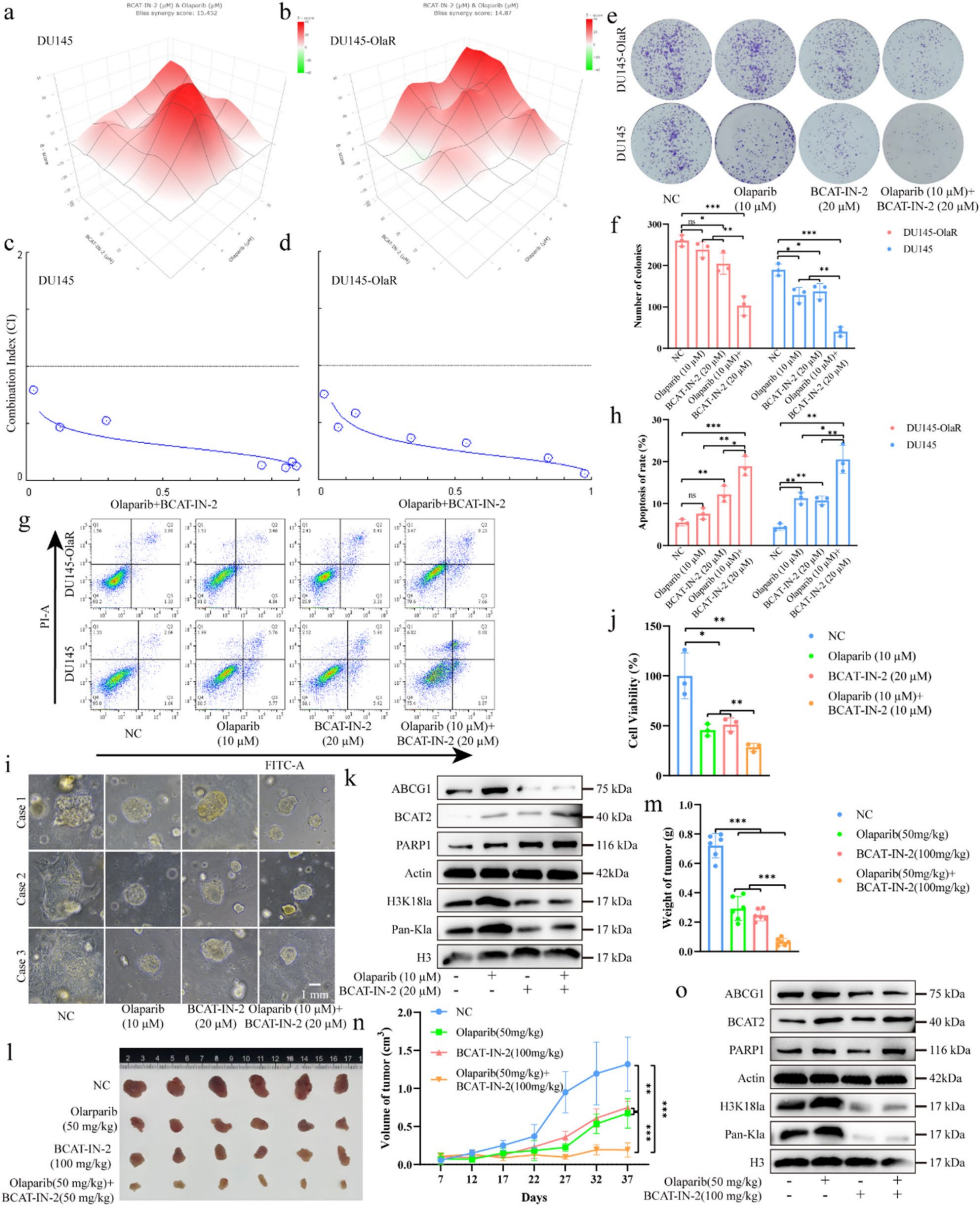
Targeted inhibition of BCAT2 sensitizes PCa to PARPi in vitro and in vivo. **a**,** b** Bliss synergy analysis of the BCAT-IN-2 and olaparib combination in DU145 (left) and DU145-OlaR (right) cells. Synergy scores were calculated using SynergyFinder. **c**,** d** Drug synergy in DU145 (left) and DU145-OlaR (right) cells. Combination index (CI) values determined using Compusyn software: CI < 1, synergy; CI = 1, additive effect; CI > 1, antagonism. **e**,** f** Colony-formation assay assessing the combinatorial effect of BCAT-IN-2 (20 µM) and olaparib (10 µM) in DU145 and DU145-OlaR cells. **g**,** h** Analysis of apoptosis by flow cytometry in DU145 and DU145-OlaR cells treated for 48 h with olaparib (10 µM), BCAT-IN-2 (20 µM), or their combination. **i**,** j** Viability of PDOs following 5-day treatment with olaparib (10 µM), BCAT-IN-2 (20 µM), or their combination. **k** Western blot analysis in PDOs treated with BCAT-IN-2 or olaparib. **l** The DU145-OlaR xenograft model was established by subcutaneous injection into the mouse left armpit. Tumor-bearing mice were then randomized into four treatment groups. **m** Tumor weight and **(n)** volume growth curves were statistically analyzed. **o** Western blot analysis in DU145-OlaR xenograft models treated with BCAT-IN-2 or olaparib

Given the role of BCAT2 in PARPi resistance, we evaluated the therapeutic potential of BCAT2 inhibition in vitro and in vivo. Combination index (CI) and synergy were assessed using CCK-8 assays and analyzed with SynergyFinder and CompuSyn. Co-treatment with the BCAT2 inhibitor BCAT-IN-2 and olaparib significantly suppressed proliferation in DU145-OlaR and other PCa cell lines. Synergy analysis showed CI values well below 1 (Fig. 6a-d, Supplementary Fig. 9a-d), indicating strong synergy between BCAT2 and PARP inhibition. To assess cellular responses, we performed colony-formation and flow-cytometric apoptosis assays following olaparib treatment. Co-treatment with a BCAT2 inhibitor and PARPi produced synergistic anti-tumor effects, with markedly reduced clonogenic survival (Fig. 6e-f, Supplementary Fig. 9e-f) and increased apoptosis (Fig. 6g-h, Supplementary Fig. 9g-h) relative to single agents.

Furthermore, in order to verify the universality of the BCAT2-PARPi axis as a therapeutic target in the PDOs and CDXs models, we used olaprib and niraparib for validation. Consistent with before findings, BCAT2 inhibition synergized with olaparib or niraparib in PDOs (Fig. 6i-j, Supplementary Fig. 10a-b) and reversed PARPi-induced ABCG1 upregulation (Fig. 6k). To test whether BCAT2 inhibition overcomes PARPi resistance in vivo, we established DU145-OlaR xenografts in nude mice. When tumors reached about 100 mm³, mice were randomized to vehicle, olaparib or niraparib (50 mg/kg), BCAT-IN-2 (100 mg/kg), or the combination, administered by oral gavage three times weekly for one month. The combination significantly reduced tumor volume and weight compared with either monotherapy (Fig. 6l-n. Supplementary Fig. 10c-e). In line with the mechanism, BCAT2 inhibition also reversed PARPi-induced ABCG1 upregulation in vivo (Fig. 6o). Collectively, these results support BCAT2 inhibition as a promising strategy to restore PARPi sensitivity in PCa.

## Discussion

**Fig. 7:**
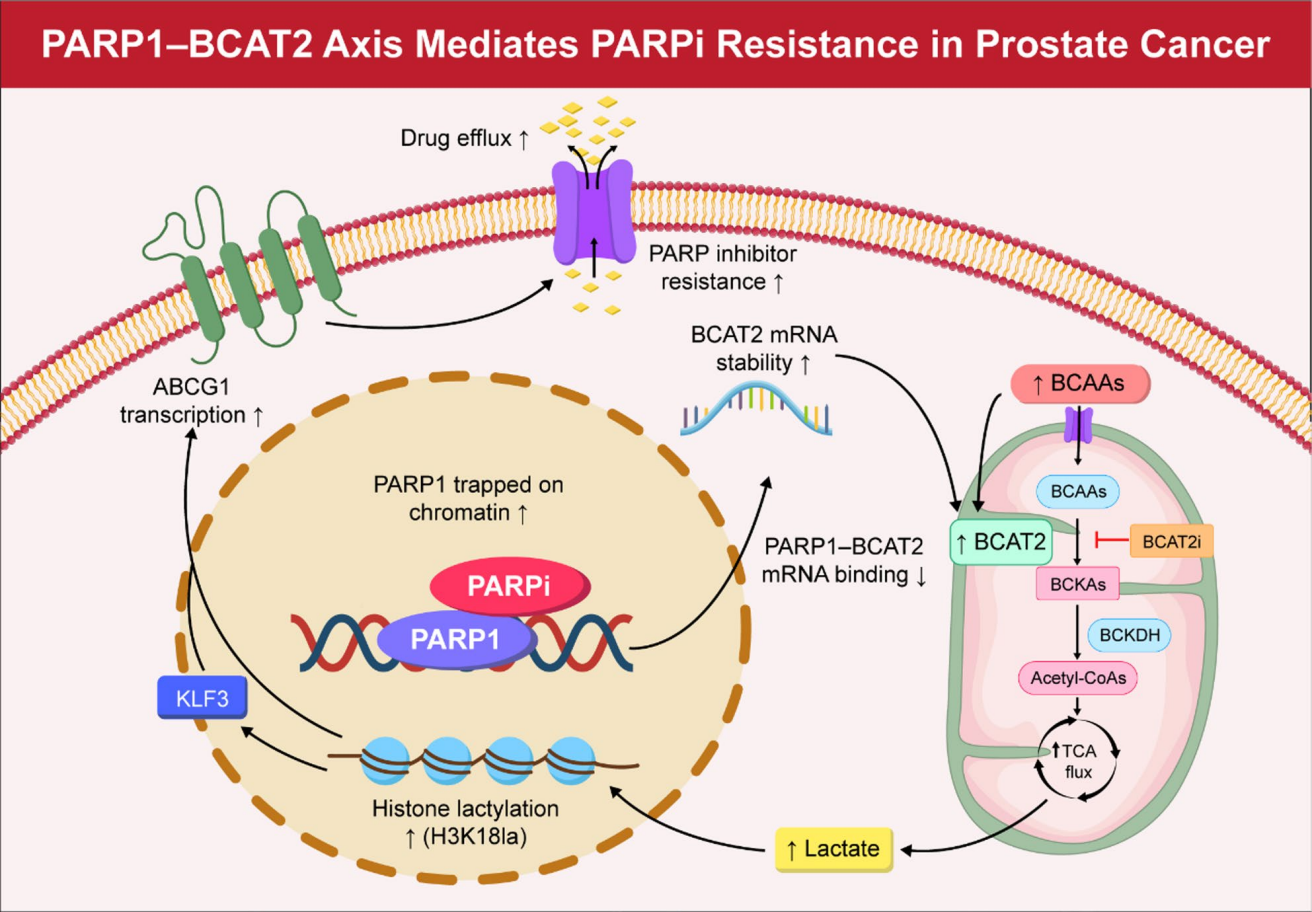
Molecular mechanism model of the role of BCAT2 in PARPi resistance. PARPi induces the capture of PARP1, reduces the mRNA binding of PARP1 and BCAT2, increases the expression of BCAT2, thereby promoting lactate secretion, leading to enhanced H3K18la level, recruiting KLF3 to jointly promote the transcription of ABCG1, resulting in drug resistance in prostate cancer cells

We define a signaling axis that drives PARPi resistance in PCa via an integrated metabolic–epigenetic mechanism. Beyond its canonical DNA repair role, PARP1 acts as an RBP that stabilizes BCAT2 mRNA, establishing a metabolic basis for resistance. BCAT2-driven BCAA catabolism increases lactate, which promotes H3K18 lactylation (H3K18la) at the ABCG1 promoter. This epigenetic change recruits KLF3 and activates ABCG1, completing a PARP1–BCAT2–H3K18la–ABCG1 circuit that reduces PARPi efficacy (Fig. 7). Notably, pharmacological BCAT2 inhibition restored PARPi sensitivity across multiple preclinical models, including patient-derived organoids and mouse xenografts, highlighting the translational promise of this target.

PARP inhibitors show activity in non–BRCA-mutant tumors, possibly through PARP1–DNA trapping [33, 34]. Although resistance mechanisms are well characterized in BRCA-mutant disease, they remain unclear in HR-proficient settings [35]. ATM deficiency leads to defective DNA damage checkpoint signaling and metabolic rewiring characterized by increased oxidative stress and dependency on alternative nutrient sources. BCAT2-mediated BCAA catabolism provides both NADPH for redox maintenance and acetyl-CoA for histone modifications essential for DNA repair gene expression. In ATM-loss cells, this metabolic dependency becomes critical. Our finding that BCAT2 inhibition simultaneously induces metabolic crisis and impairs compensatory DNA repair, creating synthetic lethality with PARPi even when HR remains functional.

Beyond transcriptional control of ABC transporters [29], evidence indicates that BCAAs and BCAT2 may participate in DNA damage repair [28]. BCAT2 could thus drive resistance via dual routes—ABCG1-mediated drug efflux and facilitation of DNA repair.

The pleiotropic effects of PARPi extend far beyond DNA repair to include broad gene regulation [36, 37]. We show that PARP1–DNA trapping during PARPi treatment reduces PARP1 binding to BCAT2 mRNA, increasing BCAT2 expression and fostering acquired resistance. This aligns with reports that PARPi modulates tumor-associated gene expression and can lower apoptotic thresholds in combinations. Consequently, evaluation of PARPi should consider DNA repair outcomes and gene-expression programs, especially under prolonged exposure when durable transcriptional changes emerge [38, 39].

PARP1 regulates gene expression as a co-regulator of DNA-binding factors, a chromatin modifier, and a mediator of DNA methylation [40, 41]. It modulates chromatin architecture largely through histone PARylation and nucleosome destabilization [42, 43]. Together, PARP1 and NASP escort and reintegrate expelled histones, limiting DNA damage and promoting PARPi resistance [44–46]. PARP1 also supports cell division by localizing to centromeres and kinetochores to govern microtubule organization and ensure the fidelity of mitosis and chromosome segregation [47, 48]. Moreover, PARP1 is a novel RBP that influences multiple RNA-processing steps and modulates mRNA to regulate gene expression [31]. PARPi efficacy depends on capturing PARP1 on chromatin [49]. Chemical inhibition or PARP1 loss can expel chromatin-bound H3–H4 in a NASP-dependent manner. Our observation that PARPi enhances BCAT2 mRNA stability via altered PARP1 binding adds a layer to PARP1-mediated regulation and its impact on therapy.

This study has some limitations. The numbers of clinical specimens and PDOs are modest; validation in large, independent cohorts is needed. Tumor-microenvironment heterogeneity also warrants study; spatial transcriptomics and single-cell RNA-seq could resolve context-dependent resistance mechanisms. While we demonstrate that BCAT-IN-2 restores PARPi sensitivity in vivo, we acknowledge that pharmacodynamic markers of target engagement—including plasma and intratumoral BCAA levels—were not assessed in this study; future investigations will incorporate these metabolic biomarkers to establish dose-response relationships and confirm on-target activity. Together, these findings support the therapeutic potential of BCAT2 inhibition, though we also note that its pharmacokinetics and safety in humans must be established before clinical translation. These constraints do not detract from our core conclusions but highlight priorities for future work to guide rational strategies against PARPi resistance. Additionally, while our current study demonstrates the BCAT2-ABCG1-PARP axis in prostate cancer, we acknowledge that both BCAT2 and ABCG1 are dysregulated in various malignancies including pancreatic, colorectal, and breast cancers, suggesting this metabolic-stemness mechanism may not be prostate-specific. Systematic investigation across multiple cancer types—including pancreatic cancer, where USP1-BCAT2 interaction was previously established—is warranted to determine the broader applicability of targeting this axis with PARP inhibitors. Future studies in other malignancies such as pancreatic and colorectal cancer, where BCAT2 is known to play oncogenic roles, will determine whether this therapeutic strategy is broadly applicable.

In conclusion, we uncover roles for PARP1 and lactate-mediated epigenetic regulation that support a compelling rationale to target BCAT2 to overcome clinical PARPi resistance. Future studies should define PARP1 structural features governing RNA binding, test whether PARP1-induced chromatin accessibility varies across genomic regions, and clarify the contribution of its enzymatic activity. Integrating metabolic and epigenetic targeting may improve management of PARPi-resistant PCa and other PARPi-treated malignancies.

## Conclusions

Our results show that PARP1 reduces BCAT2 by directly binding its mRNA, establishing a regulatory axis of PARPi resistance. The PARP1–BCAT2/H3K18la–ABCG1 pathway drives acquired resistance, and importantly, pharmacological BCAT2 inhibition restored PARPi sensitivity in prostate cancer.

## Supplementary Information

Supplementary Material 1.

## Data Availability

The data that support the findings of this study are available from the corresponding author upon reasonable request.

## Abbreviations

PARPi: Poly (ADP-ribose) polymerase inhibitor
PCa: Prostate cancer
RIP: RNA-binding protein immunoprecipitation
RIP-seq: RNA immunoprecipitation sequencing
RNA-seq: RNA sequencing
ChIP: Chromatin immunoprecipitation
CUT&Tag: Cleavage Under Targets and Tagmentation
DBD: DNA-binding domain
BCAA: branched-chain amino acid
TCA: tricarboxylic acid
RBP: RNA-binding protein
DU145-OlaR: Olaparib-resistant DU145 cells
IC₅₀: Determination of 50% inhibitory concentration
KEGG: Kyoto Encyclopedia of Genes and Genomes
IHC: Immunohistochemical
PDO: Patient-derived organoid
CBP: chromatin-binding protein
WCP: whole-cell protein
ACTD: Actinomycin D
RT-qPCR: Reverse transcription-quantitative PCR
WB: Western blot
ECAR: Extracellular acidification rate
OCR: Oxygen consumption rate
IGV: Integrative Genomics Viewer
CI: Combination index
