# Supplementary material for: PARP1-BCAT2 axis upregulates ABCG1 via histone lactylation to drive acquired PARP inhibitor resistance in prostate cancer
Source: J Exp Clin Cancer Res. 2026 May 11;45:149. doi: 10.1186/s13046-026-03719-1 (PMC13330160; doi:10.1186/s13046-026-03719-1)
Supplement: Supplementary file 1 — Supplementary Material 1. [file 13046_2026_3719_MOESM1_ESM.docx]

**Supplementary Tables**

**Supplementary Table 1: Primary antibodies list**

| Antibodies | Manufacturer | Catalog# |
| --- | --- | --- |
| Actin | Cell Signaling Technology | 4970 |
| BCAT2 | Cell Signaling Technology | 79764 |
| Caspase 3 | Cell Signaling Technology | 9664T |
| BCL2 | Abcam | ab182858 |
| BAX | Abcam | ab32503 |
| PARP1 | Cell Signaling Technology | 9532 |
| Histone H3 | Abclonal | A22348 |
| Flag | Abclonal | AE092 |
| Pan-Kla | PTMBio | PTM-1401RM |
| H3K18la | PTMBio | PTM-1427RM |
| H3K14la | PTMBio | PTM-1414RM |
| H3K56la | PTMBio | PTM-1421RM |
| ABCG1 | Abclonal | A4328 |
| LDHA | Abclonal | A21893 |
| LDHB | Abclonal | A27584 |
| KLF3 | Abcam | ab154531 |
| H2AX | Abclonal | A11412 |
| γ-H2AX | Abclonal | AP1555 |
| Ubiquitin | Abclonal | A19686 |
| CD133 | Cell Signaling Technology | 64326 |
| ALDH1 | Abclonal | **A0157** |
| KLF3 | Abcam | ab154531 |

**Supplementary Table 2: The primer sequences for RT-qPCR, PCR and CHIP**

| Gene | Forward primer 5'-3' | Reverse primer 5'-3' |
| --- | --- | --- |
| RT-Actin | CATGTACGTTGCTATCCAGGC | CTCCTTAATGTCACGCACGAT |
| RT-BCAT2 | TTACGCGCCGCACGGATCAT | GGTCGGTAAATGTCTTCCCAAAC |
| RT-PARP1 | CGGAGTCTTCGGATAAGCTCT | TTTCCATCAAACATGGGCGAC |
| RT-ABCG1 | GAGGGATTTGGGTCTGAACTGC | TCTCACCAGCCGACTGTTCTGA |
| PCR-BCAT2 | CCTGGGGTTCCGCTGC | CCCCATTAAAGCCTCGTGCT |
| CHIP-ABCG1 | TCTCCTGCCTCAGCCTACAT | CACCACTTTGGGAGGTCAGG |

**Supplementary Table 3: The sequence of the shRNAs or siRNAs**

| ShRNA |  | Sequence (5'- 3') | |
| --- | --- | --- | --- |
| shNC  shBCAT2-1 | Forward  Reverse  Forward | | GATCTGTTCTCCGAACGTGTCACGTTTCAAGAGAACGTGACACGTTCGGAGAATTTTTTC  AATTGAAAAAATTCTCCGAACGTGTCACGTTCTCTTGAAACGTGACACGTTCGGAGAACA  gatccAGGGCATGAAGGCGTTCAAAGCTCGAGCTTTGAACGCCTTCATGCCCTTTTTTT  aattAAAAAAAGGGCATGAAGGCGTTCAAAGCTCGAGCTTTGAACGCCTTCATGCCCTg |
|  | Reverse | |  |
| shBCAT2-2 | Forward  Reverse | | gatccGTGGGAACCATGAACATCTTTGCTCGAGCAAAGATGTTCATGGTTCCCATTTTTT  aattAAAAAATGGGAACCATGAACATCTTTGCTCGAGCAAAGATGTTCATGGTTCCCACg |
| shPARP1-1 | Forward | | GATCCATGACACCTCTCTACTATATACTCGAGTATATAGTAGAGAGGTGTCATTTTTTT  AATTAAAAAAATGACACCTCTCTACTATATACTCGAGTATATAGTAGAGAGGTGTCATG |
|  | Reverse | |  |
| shPARP1-2 | Forward  Reverse | | GATCCATGAAGTGGCGAAGAAGAAATCTCGAGATTTCTTCTTCGCCACTTCATTTTTTT AATTAAAAAAATGAAGTGGCGAAGAAGAAATCTCGAGATTTCTTCTTCGCCACTTCATG |
| shABCG1-1 | Forward  Reverse | | gatccGCTGCAATCTTGTGCCATATTTCTCGAGAAATATGGCACAAGATTGCAGTTTTTT  aattAAAAAACTGCAATCTTGTGCCATATTTCTCGAGAAATATGGCACAAGATTGCAGCg |
| shABCG1-2 | Forward | | gatccGTGGTGAGTTGGTGGCCATTATCTCGAGATAATGGCCACCAACTCACCATTTTTT |
|  | Reverse | | aattAAAAAATGGTGAGTTGGTGGCCATTATCTCGAGATAATGGCCACCAACTCACCACg |
| siNC |  | | UUCUCCGAACGUGUCACGU |
| siKLF3 |  | | ACCCTAGAAGGTTTAACTAAAAG |
| siLDHA |  | | GGCAAAGACUAUAAUGUAA |
| siLDHB |  | | GCGUUAUCAACCAGAAGCUAA |

**Supplementary Figures**


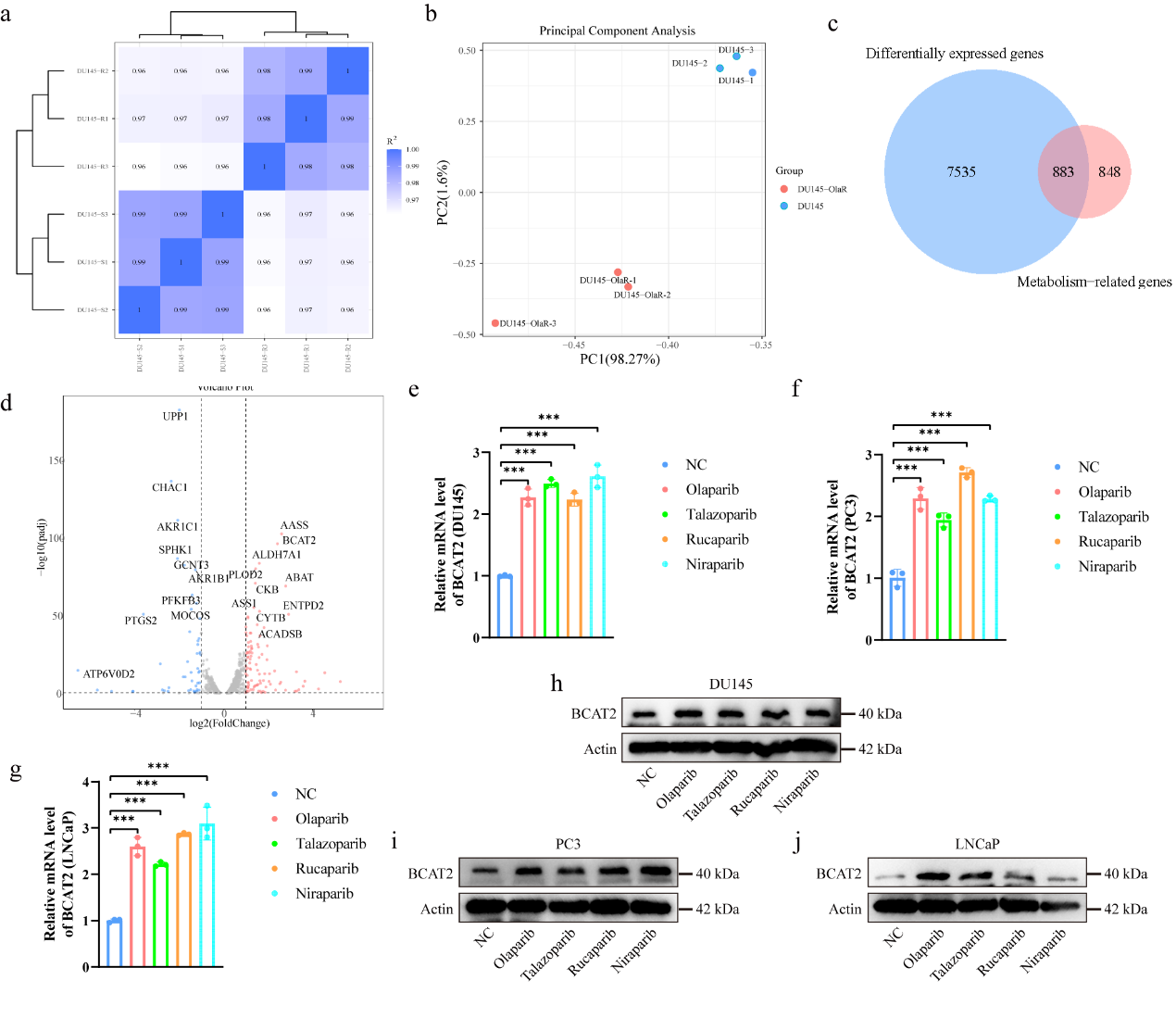


**Supplementary Fig. 1. (a)** Pearson’s correlation analysis between the mRNA expression profiles of the DU145 (S) (n = 3) and DU145-OlaR (R) (n = 3) samples. **(b)** Principal component analysis between the two groups by RNA-seq. **(c)** Venn diagram of the overlap between the RNA-seq data and metabolism-related genes. **(d)** Volcano map of differentially expressed metabolism-related genes between DU145 and DU145-OlaR groups. BCAT2 mRNA levels in **(e)** DU145, **(f)** PC3, and **(g)** LNCaP cells with PARPi treatment for 48 h (Olaparib: 10 μM; Talazoparib: 100 nM; Rucaparib: 10 μM; Niraparib: 10 μM). BCAT2 protein expression levels in **(h)** DU145, **(i)** PC3, and **(j)** LNCaP cells with PARPi treatment for 48 h (Olaparib: 10 μM; Talazoparib: 100 nM; Rucaparib: 10 μM; Niraparib: 10 μM).


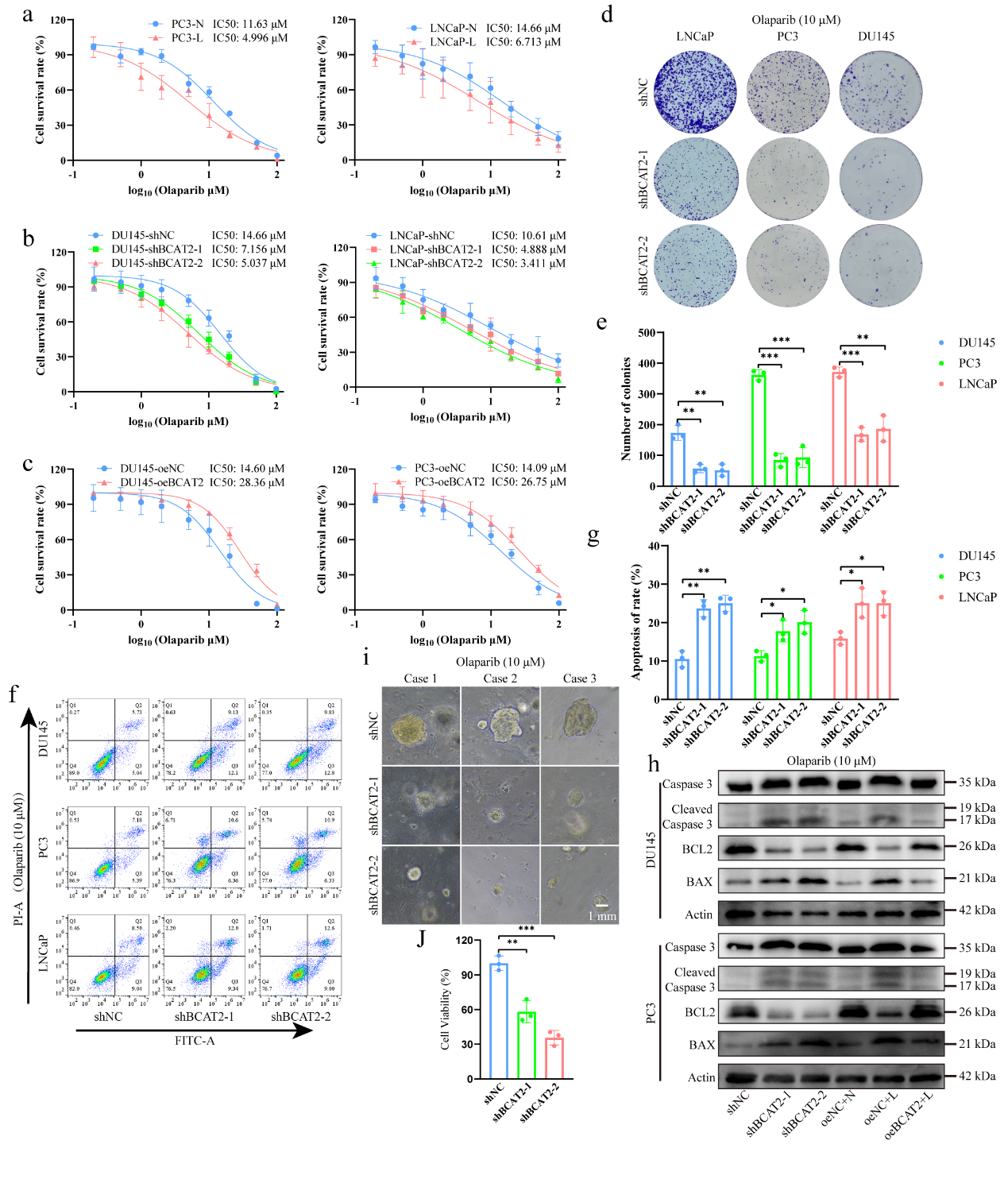


**Supplementary Fig. 2. (a)** Olaparib IC_50_ curves in PCa cells under low BCAA (-L) vs. normal (-N) culture conditions. **(b)** Olaparib IC_50_ curves in shBCAT2 PCa cells. **(c)** Olaparib IC_50_ curves in oeBCAT2 PCa cells. **(d, e)** Clonogenic assays of PCa cells in the presence of PARPi for 10-14 days (Olaparib: 10 μM). **(f, g)** Apoptotic rate of PCa cells treated with Olaparib (10 µM) for 48 h. (**h**) WB analysis of apoptosis-related protein levels in DU145 and PC3 cells, treated with Olaparib (10 µM) for 48 h. (**i, j**) The effect of BCAT2 knockdown on the sensitivity of PDOs to Olaparib. Organoids were treated with 10 µM olaparib for 5 days.


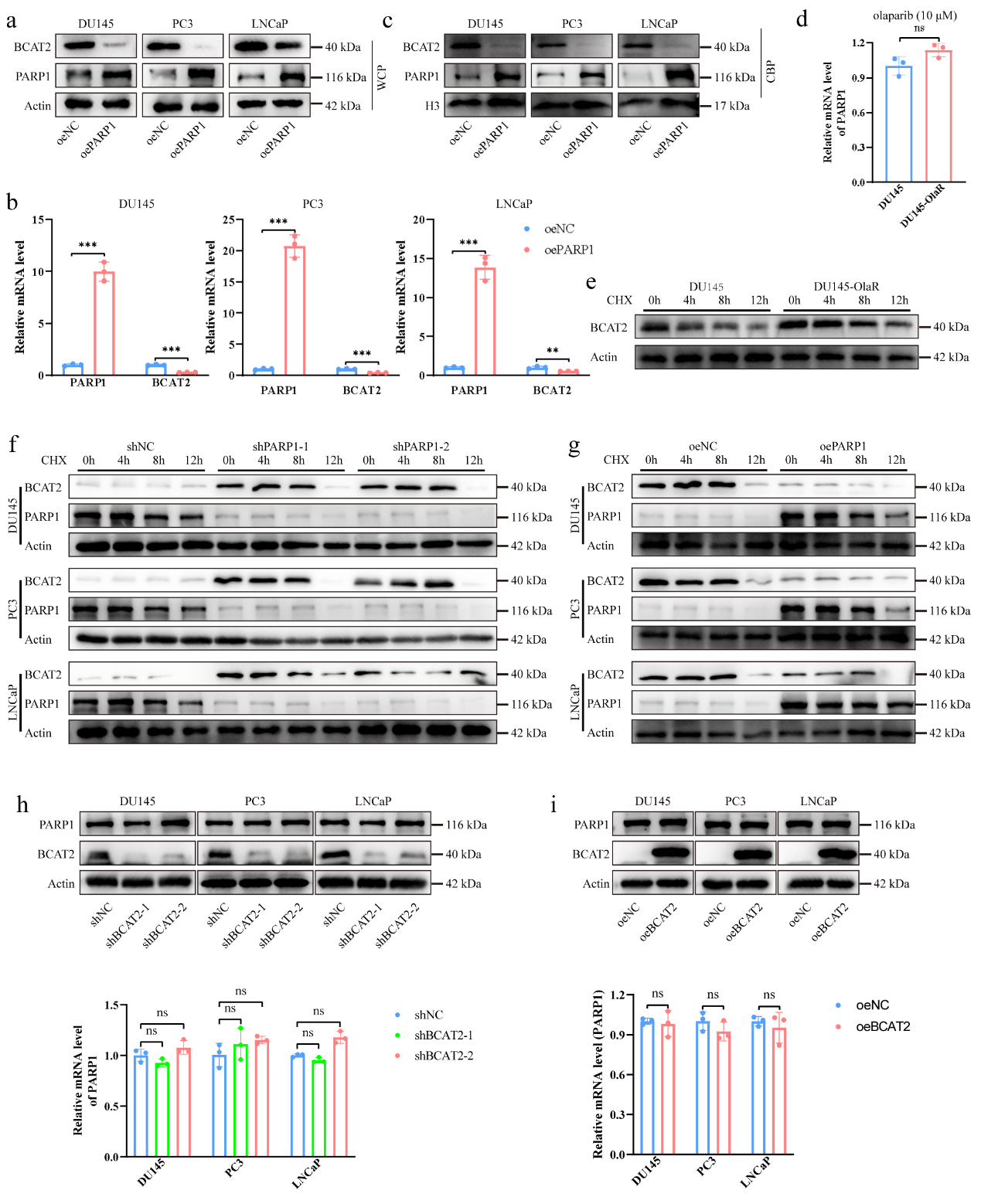


**Supplementary Fig. 3. (a)** Western blot analysis of the whole-cell protein (WCP) in PARP1 overexpression PCa cells. **(b)** RT-qPCR analysis of the BCAT2 and PARP1 mRNA levels in PARP1 overexpression PCa cells. **(c)** Western blot analysis of the chromatin-binding protein (CBP) in PARP1 overexpression PCa cells. **(d)** PARP1 mRNA expression levels in DU145 and DU145-OlaR cells treated with 10 μM olaparib. **(e)** The PCBP1 protein level was detected in DU145 and DU145-OlaR cells. The cells were treated with 100 μg/ml cycloheximide (CHX, MedChemexpress, USA) for a specified time. The PCBP1 protein level was detected in PARP1 **(f)** knockdown or **(g)** overexpression cells. **(h)** Western blot and RT-qPCR analysis in BCAT2 knockdown PCa cells. **(i)** Western blot and RT-qPCR analysis in BCAT2 overexpression PCa cells.


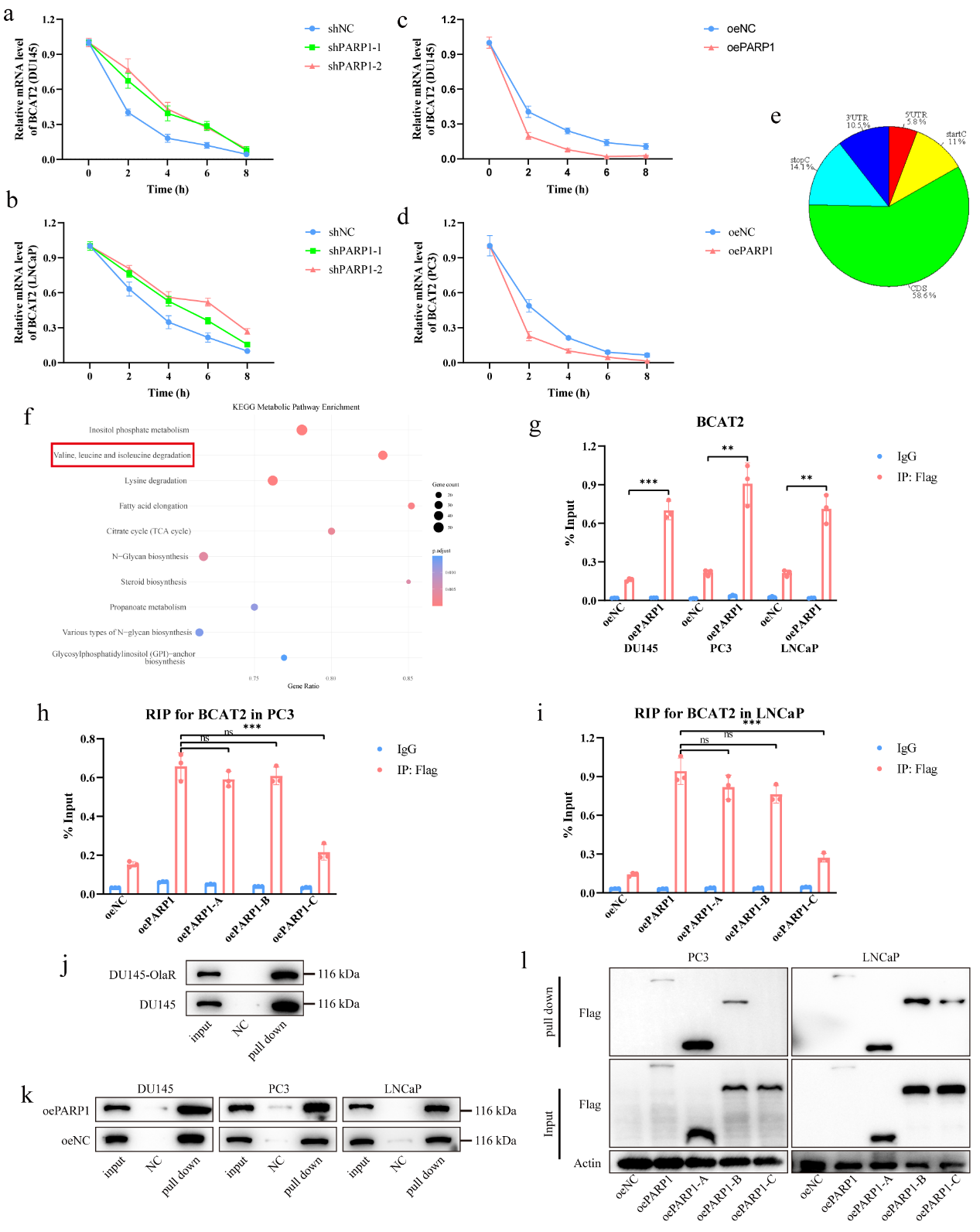


**Supplementary Fig. 4. (a-d)** The BCAT2 mRNA level was detected in PCa cells. The cells were treated with 4 μM actinomycin D (ACTD, MedChemExpress, USA) for a specified time. **(e)** The distributions of PARP1-binding regions are shown in the pie charts. **(f)** KEGG enrichment analysis of PARP1-bound RNAs. **(g)** RIP-qPCR was used to examine RNA enrichment in PARP1 overexpression cells. **(h, i)** RIP-qPCR analysis in PC3 and LNCaP cells transfected with various truncated mutants. **(j)** RNA pull-down assays in DU145 and DU145-OlaR cells. **(k)** RNA pull-down assays in PARP1 overexpression PCa cells. **(l)** RNA–protein pull-down assays in PC3 and LNCaP cells transfected with various truncated mutants.


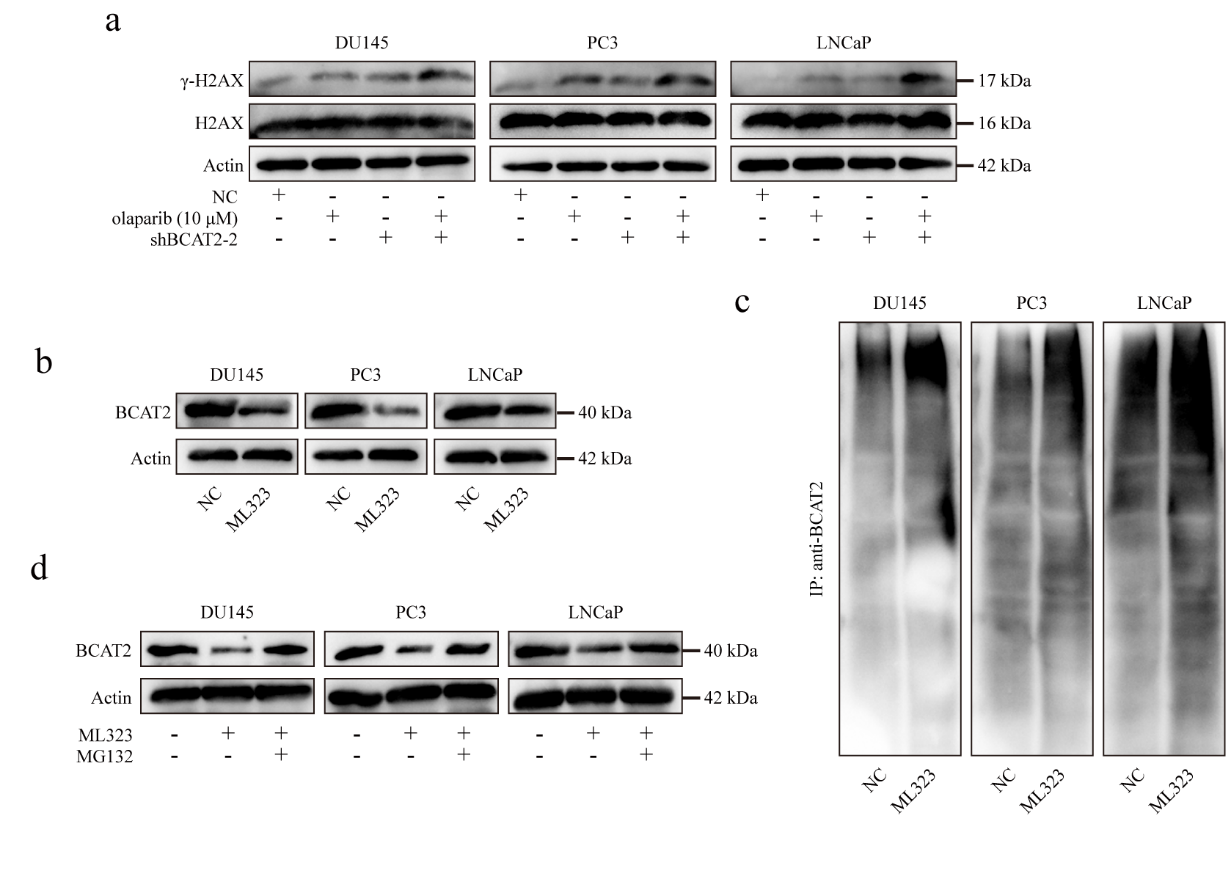


Supplementary Fig. 5. (a) Western blot analysis of γ-H2AX, and H2AX levels in PCa cells upon treatment with olaparib (10 µM, 48 h) or BCAT2 knockdown. (b) WB analysis of BCAT2 in PCa cells upon treatment with ML323 (10 µM, 48 h). (c) WB verified the effect of ML323 on ubiquitination level of BCAT2. (d) WB analysis of BCAT2 in PCa cells upon treatment with ML323 (10 µM, 48 h) or MG132 (10 μM, 4 h).


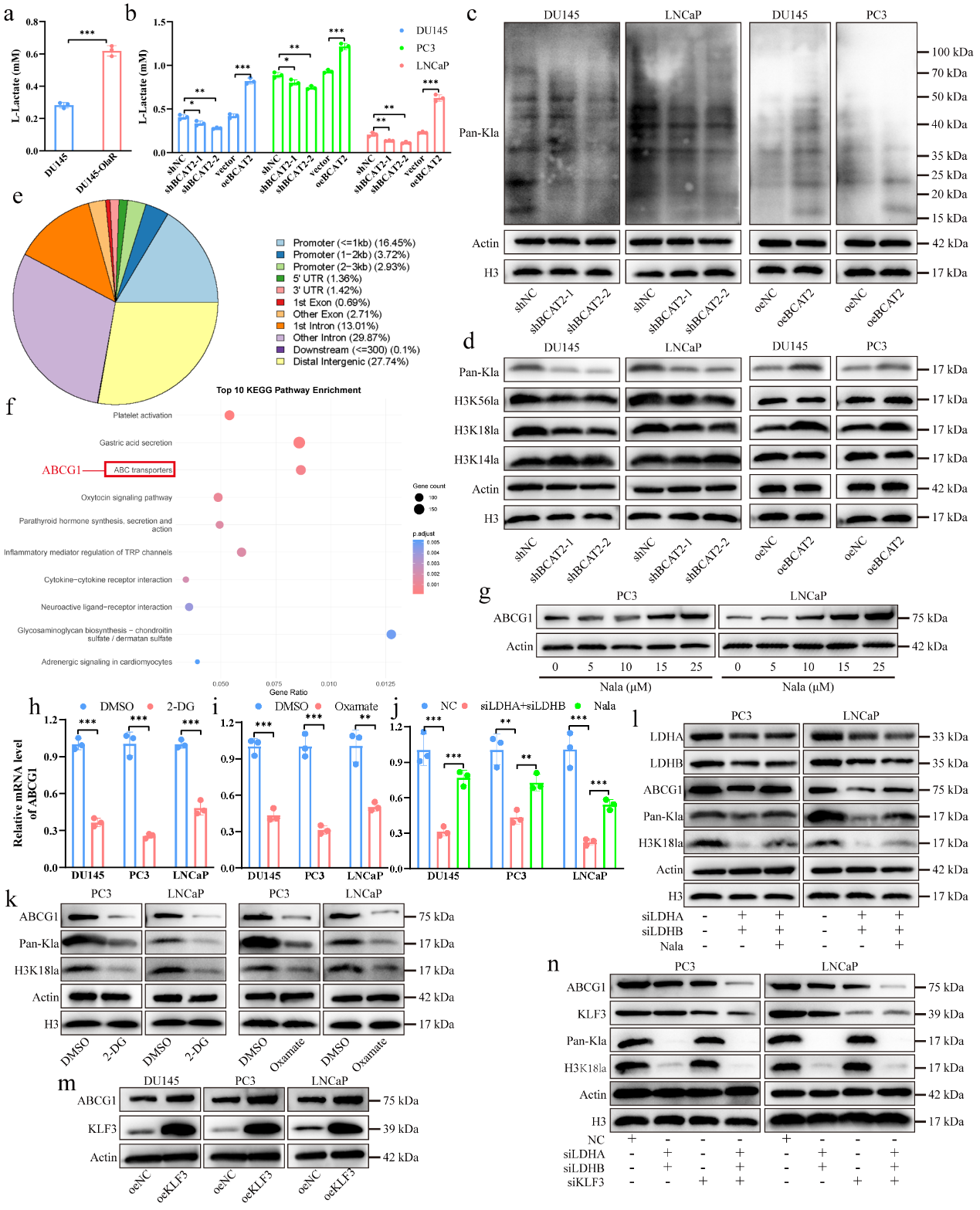


**Supplementary Fig. 6. (a)** Measurement of L-lactate concentration in DU145 or DU145-OlaR cells. **(b)** Measurement of L-lactate concentration in PCa cells treated with BCAT2 knockdown or overexpression. **(c)** Western blotting analysis of global lactylation levels in PCa cells. **(d)** Western blotting analysis of some histone lactylation sites’ levels in PCa cells. **(e)** Distribution ratio of H3K18la sites across the gene sequences. **(f)** KEGG enrichment analysis revealed the significantly enriched items based on the H3K18la signature. **(g)** Western blotting analysis of ABCG1 level in PC3 and LNCaP cells treated with different concentrations of sodium lactate (Nala) for 48 h. **(h-j)** RT-qPCR data showing ABCG1 expression in PCa cells upon treatment with histone lactylation inhibitors (2-DG or Oxamate) or LDHA/B inhibition. **(k)** Western blot analysis of ABCG1, Pan-Kla, and H3K18la levels in PC3 and LNCaP cells upon histone lactylation inhibitors (2-DG or Oxamate). **(l)** Western blot analysis of ABCG1, Pan-Kla, and H3K18la levels in DU145 cells upon LDHA/B inhibition. **(m)** Western blot analysis in KLF3 overexpression PCa cells. **(n)** Western blot analysis of ABCG1 levels in PC3 and LNCaP cells upon LDHA/B inhibition or KLF3 knockdown.


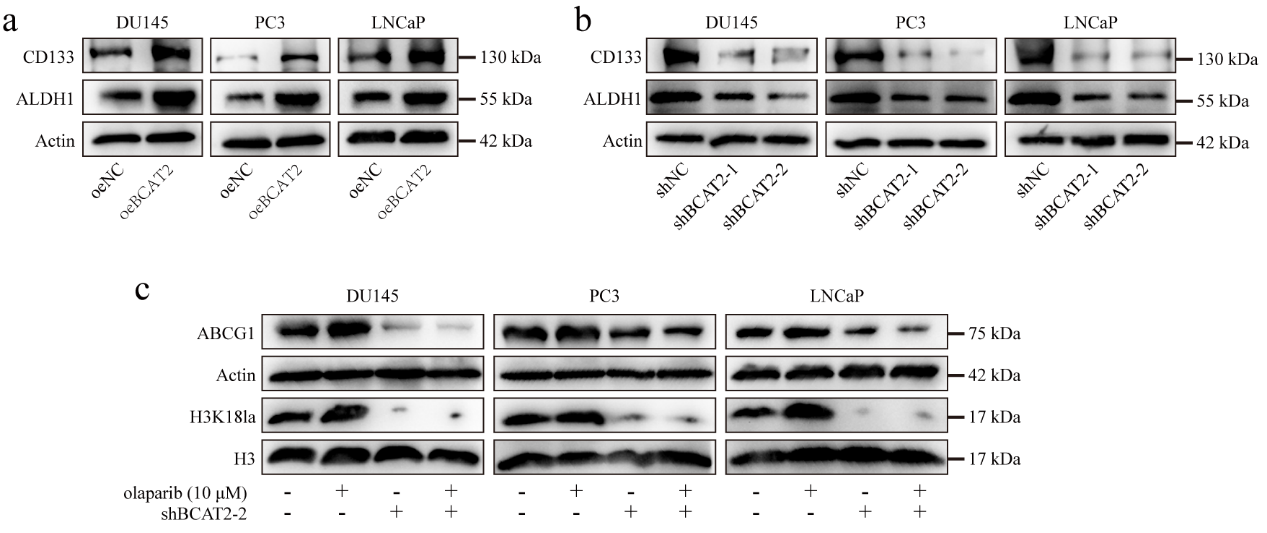


Supplementary Fig. 7. (a) WB analysis of cancer stemness markers in BCAT2 overexpression PCa cells. (b) WB analysis of cancer stemness markers in BCAT2 knockdown PCa cells. (c) WB analysis in PCa cells treated with olaparib (10 µM, 48 h) or BCAT2 knockdown.


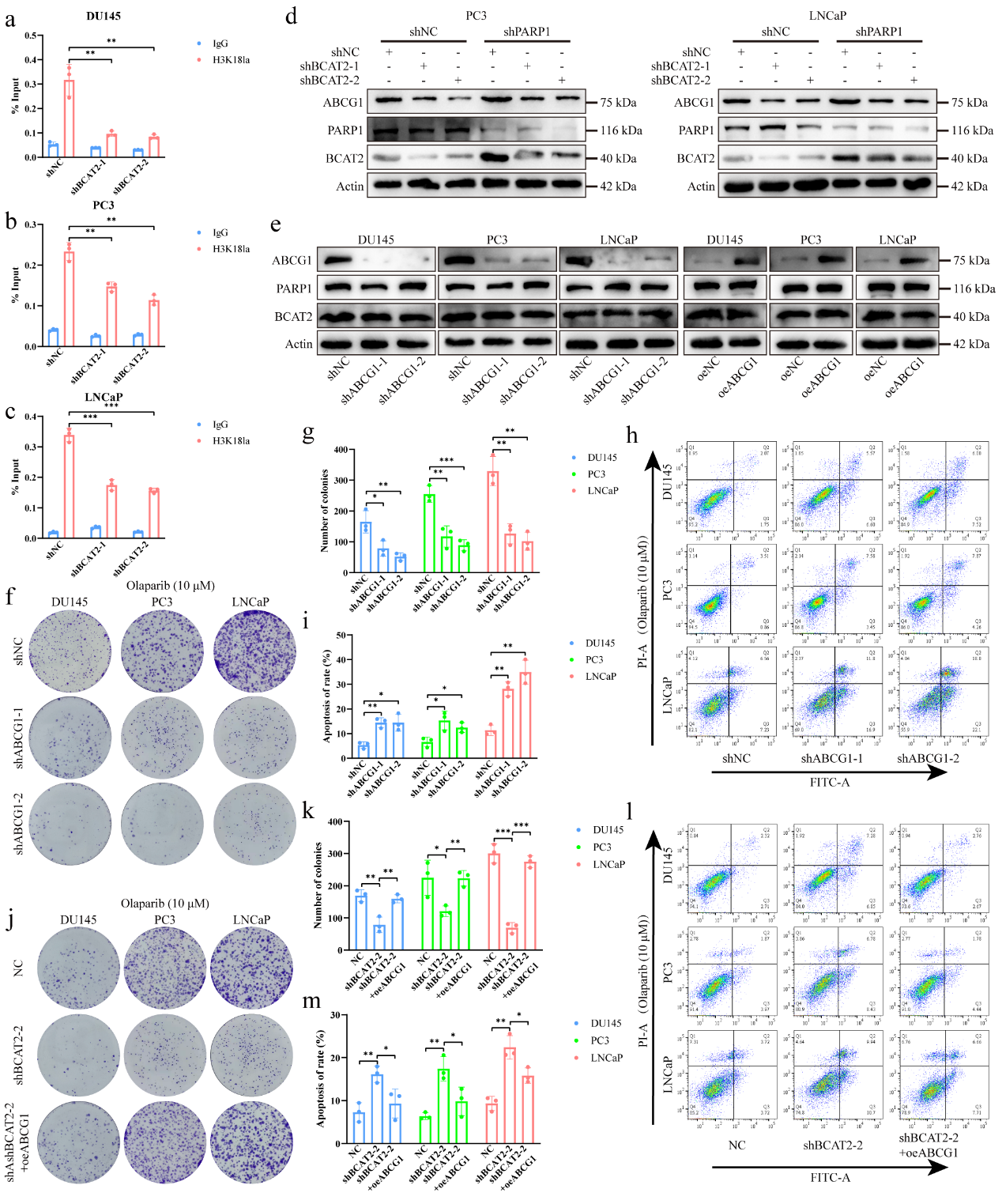


**Supplementary Fig. 8. (a-c)** ChIP–qPCR assay of H3K18la status in the ABCG1 genomic region in BCAT2 knockdown cells. **(d)** Western blotting analysis in shNC- and shPARP1-PCa cells, which were transfected with shNC or shBCAT2, respectively. **(e)** Western blot analysis of BCAT2 and PARP1 levels in PCa cells upon ABCG1 knockdown or overexpression. **(f, g)** Clonogenic assays of ABCG1 knockdown cells in the presence of 10 μM olaparib for 10-14 days. **(h, i)** Apoptotic rate of ABCG1 knockdown cells treated with olaparib (10 µM) for 48 h. **(j, k)** Clonogenic assays of PCa cells in the presence of 10 μM olaparib for 10-14 days. **(l, m)** Apoptotic rate of PCa cells treated with Olaparib (10 µM) for 48 h.


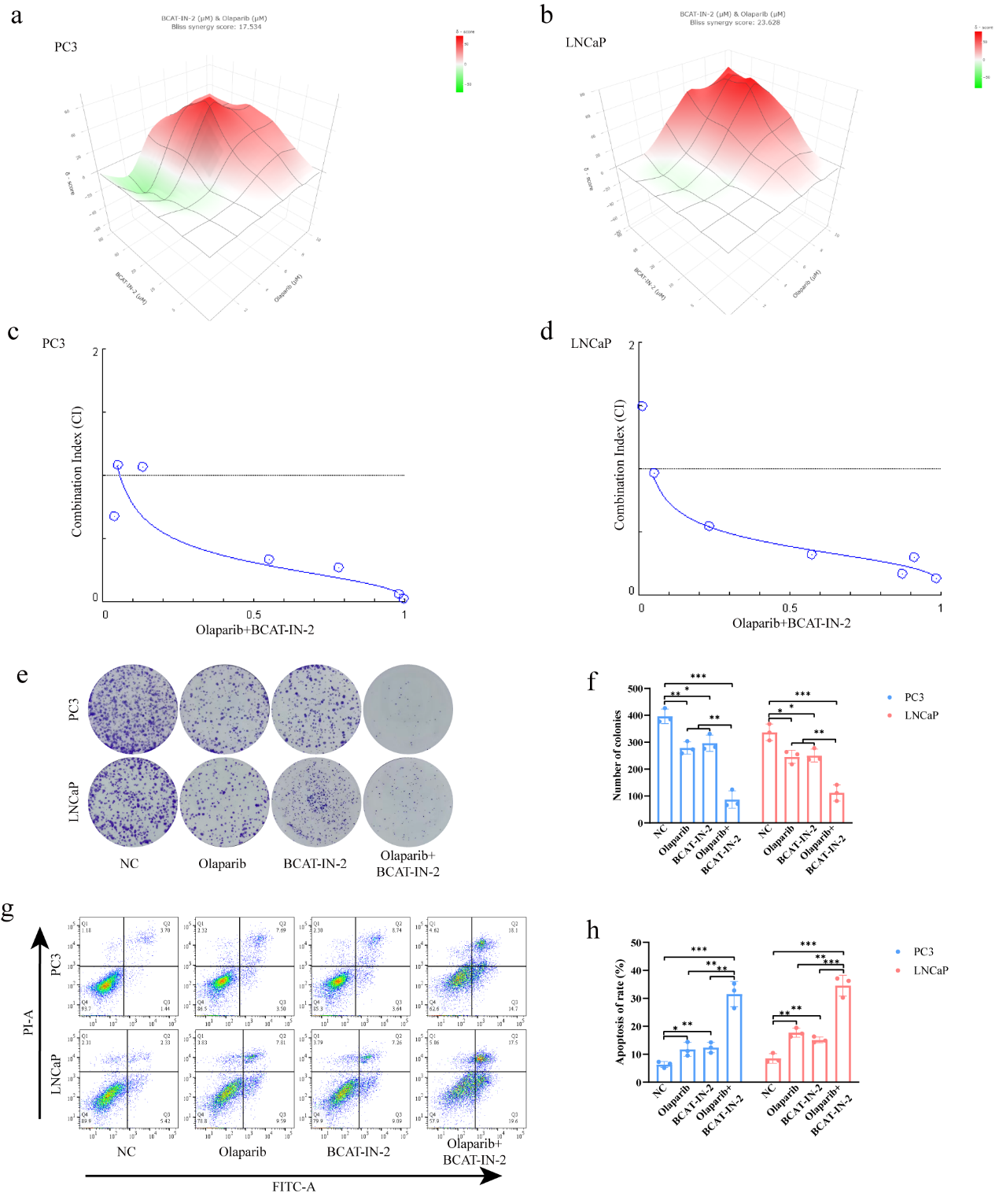


**Supplementary Fig. 9. (a, b)** Bliss synergy analysis of the BCAT-IN-2 and Olaparib combination in PC3 (left) and LNCaP (right) cells. Synergy scores were calculated using SynergyFinder. **(c, d)** Drug synergy in PC3 (left) and LNCaP (right) cells. Combination index (CI) values determined using Compusyn software: CI < 1, synergy; CI = 1, additive effect; CI > 1, antagonism. **(e, f)** Colony-formation assay assessing the combinatorial effect of BCAT-IN-2 (20 µM) and Olaparib (10 µM) in PC3 and LNCaP cells. **(g, h)** Flow-cytometric analysis of apoptosis in PC3 and LNCaP cells after 48 h of treatment with olaparib (10 µM), BCAT-IN-2 (20 µM), or their combination.


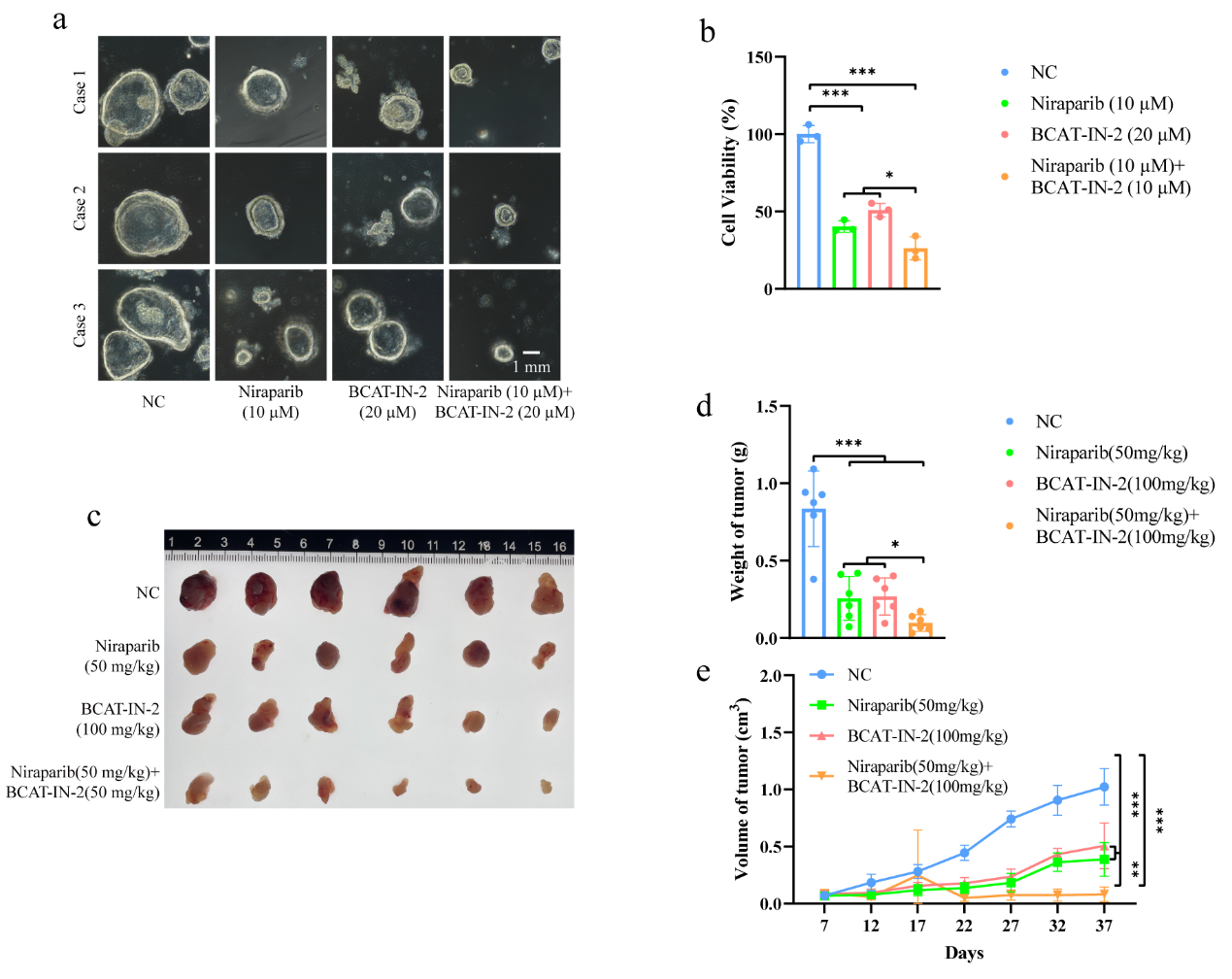


Supplementary Fig. 10. (a, b) Viability of PDOs following 5-day treatment with niraparib (10 µM), BCAT-IN-2 (20 µM), or their combination. (c) The DU145-OlaR xenograft model was generated and randomly assigned to four treatment cohorts. (d) Tumor mass and (e) tumor growth kinetics were analyzed statistically.
